# High-quality genome of black wolfberry (*Lycium ruthenicum* Murr.) provides insights into the genetics of anthocyanin biosynthesis regulation

**DOI:** 10.1093/hr/uhae298

**Published:** 2024-10-23

**Authors:** Yuhui Xu, Haoxia Li, Tongwei Shi, Qing Luo, Yuchao Chen, Shenghu Guo, Weiwei Tian, Wei An, Jian Zhao, Yue Yin, Jun He, Rui Zheng, Xiaojie Liang, Yajun Wang, Xiyan Zhang, Zhigang Shi, Linyuan Duan, Xiaoya Qin, Ting Huang, Bo Zhang, Ru Wan, Yanlong Li, Youlong Cao, Hui Liu, Sheng Shu, Aisheng Xiong, Jianhua Zhao

**Affiliations:** National Wolfberry Engineering Research Center/Wolfberry Science Research Institute, Ningxia Academy of Agriculture and Forestry Sciences, Yinchuan 750002, China; Institute of Forestry and Grassland Ecology, Ningxia Academy of Agriculture and Forestry Sciences, Yinchuan 750002, China; Planttech technologies Co. Ltd., Beijing, China; National Wolfberry Engineering Research Center/Wolfberry Science Research Institute, Ningxia Academy of Agriculture and Forestry Sciences, Yinchuan 750002, China; Agricultural Biotechnology Centre, Ningxia Academy of Agriculture and Forestry Sciences, Yinchuan 750002, China; Agricultural Biotechnology Centre, Ningxia Academy of Agriculture and Forestry Sciences, Yinchuan 750002, China; Sichuan Academy of Chinese Medical Sciences, Chengdu 610041, China; National Wolfberry Engineering Research Center/Wolfberry Science Research Institute, Ningxia Academy of Agriculture and Forestry Sciences, Yinchuan 750002, China; National Wolfberry Engineering Research Center/Wolfberry Science Research Institute, Ningxia Academy of Agriculture and Forestry Sciences, Yinchuan 750002, China; National Wolfberry Engineering Research Center/Wolfberry Science Research Institute, Ningxia Academy of Agriculture and Forestry Sciences, Yinchuan 750002, China; National Wolfberry Engineering Research Center/Wolfberry Science Research Institute, Ningxia Academy of Agriculture and Forestry Sciences, Yinchuan 750002, China; Key Laboratory of Ministry of Education for Protection and Utilization of Special Biological Resources in Western China; College of Life Science, Ningxia University, Yinchuan 750021, China; National Wolfberry Engineering Research Center/Wolfberry Science Research Institute, Ningxia Academy of Agriculture and Forestry Sciences, Yinchuan 750002, China; National Wolfberry Engineering Research Center/Wolfberry Science Research Institute, Ningxia Academy of Agriculture and Forestry Sciences, Yinchuan 750002, China; National Wolfberry Engineering Research Center/Wolfberry Science Research Institute, Ningxia Academy of Agriculture and Forestry Sciences, Yinchuan 750002, China; National Wolfberry Engineering Research Center/Wolfberry Science Research Institute, Ningxia Academy of Agriculture and Forestry Sciences, Yinchuan 750002, China; National Wolfberry Engineering Research Center/Wolfberry Science Research Institute, Ningxia Academy of Agriculture and Forestry Sciences, Yinchuan 750002, China; National Wolfberry Engineering Research Center/Wolfberry Science Research Institute, Ningxia Academy of Agriculture and Forestry Sciences, Yinchuan 750002, China; National Wolfberry Engineering Research Center/Wolfberry Science Research Institute, Ningxia Academy of Agriculture and Forestry Sciences, Yinchuan 750002, China; National Wolfberry Engineering Research Center/Wolfberry Science Research Institute, Ningxia Academy of Agriculture and Forestry Sciences, Yinchuan 750002, China; National Wolfberry Engineering Research Center/Wolfberry Science Research Institute, Ningxia Academy of Agriculture and Forestry Sciences, Yinchuan 750002, China; National Wolfberry Engineering Research Center/Wolfberry Science Research Institute, Ningxia Academy of Agriculture and Forestry Sciences, Yinchuan 750002, China; National Wolfberry Engineering Research Center/Wolfberry Science Research Institute, Ningxia Academy of Agriculture and Forestry Sciences, Yinchuan 750002, China; State Key Laboratory of Crop Genetics & Germplasm Enhancement and Utilization, Ministry of Agriculture and Rural Affairs Key Laboratory of Biology and Germplasm Enhancement of Horticultural Crops in East China, College of Horticulture, Nanjing Agricultural University, Nanjing 210095, China; State Key Laboratory of Crop Genetics & Germplasm Enhancement and Utilization, Ministry of Agriculture and Rural Affairs Key Laboratory of Biology and Germplasm Enhancement of Horticultural Crops in East China, College of Horticulture, Nanjing Agricultural University, Nanjing 210095, China; State Key Laboratory of Crop Genetics & Germplasm Enhancement and Utilization, Ministry of Agriculture and Rural Affairs Key Laboratory of Biology and Germplasm Enhancement of Horticultural Crops in East China, College of Horticulture, Nanjing Agricultural University, Nanjing 210095, China; National Wolfberry Engineering Research Center/Wolfberry Science Research Institute, Ningxia Academy of Agriculture and Forestry Sciences, Yinchuan 750002, China

## Abstract

Black wolfberry (*Lycium ruthenicum* Murr.) is an important plant for ecological preservation. In addition, its fruits are rich in anthocyanins and have important edible and medicinal value. However, a high-quality chromosome-level genome for this species is not yet available, and the regulatory mechanisms involved in the biosynthesis of anthocyanins are unclear. In this study, haploid material was used to assemble a high-quality chromosome-level reference genome of *Lycium ruthenicum*, resulting in a genome size of 2272 Mb with contig N50 of 92.64 Mb, and 38 993 annotated gene models. In addition, the evolution of this genome and large-scale variations compared with the Ningxia wolfberry *Lycium barbarum* were determined. Importantly, homology annotation identified 86 genes involved in the regulatory pathway of anthocyanin biosynthesis, five of which [*LrCHS1* (evm.TU.Chr05.295), *LrCHS2* (evm.TU.Chr09.488), *LrAOMT* (evm.TU.Chr09.809), *LrF3’5’H* (evm.TU.Chr06.177), and *LrAN2.1* (evm.TU.Chr05.2618)] were screened by differential expression analysis and correlation analysis using a combination of transcriptome and metabolome testing. Overexpression of these genes could significantly up- or downregulate anthocyanin-related metabolites. These results will help accelerate the functional genomic research of *L. ruthenicum*, and the elucidation of the genes involved in anthocyanin synthesis will be beneficial for breeding new varieties and further exploring its ecological conservation potential.

## Introduction

Black wolfberry (*Lycium ruthenicum* Murr.), an important wild edible and medicinal plant resource, is a perennial shrub belonging to the Solanaceae family. It is mainly distributed in saline–alkali desert areas in Xinjiang, Ningxia, Gansu, and Qinghai provinces in China [1]. *Lycium ruthenicum* has strong adaptability and is an excellent resource for soil and water conservation in northwest China. It also has ecological value, such as serving as a windbreak and aiding sand fixation and soil improvement, as well as being used as an alkaline soil indicator and pioneer species. The fruits of *L. ruthenicum* are rich in active compounds, such as polysaccharides, flavonoids, vitamins, and proteins [2–4], which have high nutritional and medicinal value, as well as antioxidant and antiaging [5], hypolipidemic [6], anticancer [7], and antifatigue effects [8], in addition to potential for gastrointestinal protection [9] and radiation resistance [10]. In addition, *L. ruthenicum* contains levels of anthocyanins that are higher than those of either blackcurrant or blueberry [116]. Anthocyanins have biological activities and can be used to prevent and treat various diseases [11, 12]; they are also widely used in medicines and foods, and as natural food colorants [2, 13]. Besides their medicinal value, anthocyanins have important roles in facilitating plant reproduction and protecting plants from biotic and abiotic stresses [14–16].

Anthocyanins are a class of water-soluble natural pigments specific to plants. Currently, over 700 different anthocyanins have been identified in plants [15]. Of these, the most common are cyanidin, pelargonidin, delphinidin, peonidin, petunidin, and malvidin [17, 18]. Proanthocyanidins are a type of flavonoid that are synthesized in the cytoplasm of plant cells and then modified by various processes, such as hydroxylation, glycosylation, methylation, and acylation, on the intracellular membrane to form chemically stable anthocyanidin glycosides. These then enter the vacuole via extracellular vesicles and transport proteins, where they accumulate [19]. Anthocyanins are a significant component of the fruits of *L. ruthenicum,* where 37 types have been identified, of which petunidin-3-O-rutinoside (trans-p-coumaroyl)-5-O-glucoside (PRG) is the most dominant [20, 21]. However, few studies have investigated gene function in the anthocyanin biosynthesis pathway in *L. ruthenicum*.

Anthocyanidin biosynthesis is a well-known branch of the flavonoid synthetic pathway [22–24]. The genes involved in anthocyanin biosynthesis are regulated by various factors [14, 25]. For example, MYB [26–28], bHLH/SlAN2-like [29, 30], WRKY [31], NAC [32], WD40 [33], and MADS [34] can act as transcriptional activation or suppression factors to interact with structural genes. Moreover, the co-regulation of the transcription factors (TFs) MYBA and MYBPA is involved anthocyanin biosynthesis in blue-colored berries [35]. In addition, MYB can form an MBW complex with bHLH and WD40 proteins to regulate the transcription and expression of structural genes, thereby regulating anthocyanin synthesis [14, 36, 37]. Further investigation of pepper suggested that *CaANT1*, *CaANT2*, *CaAN1*, and *CaTTG1* form an MMBW transcription complex to activate anthocyanin accumulation [38]. Methylation of MYB genes [26] and genome DNA methylation [39] are also involved in the regulation of anthocyanins. Recently, miRNAs were also reported to participate in the regulation of anthocyanin biosynthesis by targeting SPL genes [16] or *MdMYB9* and *MdMYBPA1* [40]. In addition, under some circumstances, hormones have been reported to be involved in the coordinated regulation of anthocyanins. For example, jasmonic acid mediates the JAZ1-TRB1-MYB9 complex to induce the biosynthesis of anthocyanins and proanthocyanidins in apples [41]. Abscisic acid (ABA) strongly induces the expression of *MdNAC1*, which can interact with bZIP-type TFs to promote anthocyanin synthesis in red-fleshed apples [32]. Ethylene can inhibit the expression of *PpMYB10* and *PpMYB114* through TF inhibitors, ultimately reducing anthocyanin biosynthesis [42].

In *L. ruthenicum*, previous work showed that the PRG content is lowest in green fruits but increases as the fruit ripens; in addition, the expression of *LrAN2* in fruits is significantly positively correlated with the PRG content, suggesting that this gene is involved in PRG accumulation [118]. A total of 25 significantly differentially expressed structural genes have been identified (including *PAL*, *C4H*, *4CL*, *CHS*, *CHI*, *F3H*, *F3’H*, *F3’5’H*, *DFR*, *ANS*, and *UFGT*) that might be associated with anthocyanin biosynthesis. Furthermore, several TFs, including MYB, bHLH, WD40, NAC, WRKY, bZIP, and MADS, correlate with these structural genes, suggesting their important interaction with anthocyanin biosynthesis-related genes [43]. Moreover, methyl transferase, UFGT, and BAHD are involved in the modification of synthesized anthocyanins, resulting in more chemically stable anthocyanins [44]. Two functional MYB TFs, AN2 alleles from *L. ruthenicum* and the Ningxia wolfberry *Lycium barbarum*, were identified as functional MYB TFs involved in anthocyanin biosynthesis regulation [45]. The *LrAN2*-like TF interacts with LrAN1b and LrAN11 to form an MBW complex that regulates the promoter of downstream target genes *LrDFR* and *LrANS* to control anthocyanin synthesis. When anthocyanin accumulation is too high during the later stages of fruit development, the MBW complex activates the *LrMYB3* and *LrETC1* suppressor genes to achieve feedback inhibition [31].

The genome of *L. barbarum* has been published [46]. However, the anthocyanin content in its fruit is extremely low [47]. Despite many insights into anthocyanin biosynthesis and its regulation, the absence of a high-quality reference genome is a barrier to systematic studies on the biosynthetic pathways and associated regulatory mechanisms in *L. ruthenicum*. Thus, this study used pollen-cultured haploid plants to obtain a high-quality assembly of the *L. ruthenicum* genome, followed by comparative genomic analysis to explore the genome evolution status in Solanaceae. Particularly, analyzing the genomic synteny and variations between *L. ruthenicum* and *L. barbarum* allows for further genome evolutionary conservation and functional divergence in *Lycium*. Finally, multiomics analysis and gene function verification were then combined to determine the genes and regulatory factors involved in anthocyanin biosynthesis. This study provides new genomics resources for the study of *Lycium* species, as well as novel insights into the genetics of anthocyanin biosynthesis in *L. ruthenicum*.

**Table 1:** Genome assembly and annotation overview.

Category	**Value**
Counts of scaffold sequences	580
Length of scaffold sequences (bp)	2 272 054 902
Largest scaffold length (bp)	215 860 124
Contig N50 (bp)	92 643 068
Scaffold N50 (bp)	188 110 659
GC content (%)	38.66
Ratio of sequences ordered and oriented by Hi-C (%)	97.46
Predicted gene models	38 993
Average exons per gene	4.61
Average coding sequence length (bp)	947.3
Average gene length (bp)	4069.90
COG-annotated gene models	7748
GO-annotated gene models	21 165
KEGG-annotated gene models	9304
Swiss–Prot-annotated gene models	17 115
Nr-annotated gene models	35 261
Total annotated gene models	35 294

## Results

### Genome assembly, annotation, and quality evaluation

**Figure 1:**
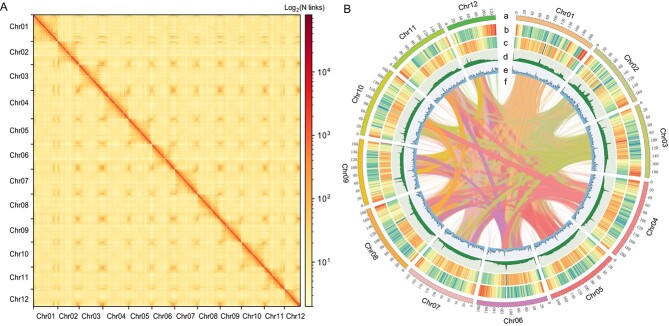
Hi-C interaction heat map and Circos plot for the genome assembly of black wolfberry (*L. ruthenicum*). (A) The interaction links of Hi-C data at a bin size of 200 kb. The interaction intensity is indicated using colors ranging from the perimeter to the diagonal, denoting the frequency of Hi-C interaction from low to high, respectively. (B) Circos plot of the genome landscape of *L. ruthenicum* with a window size of 500 kb. This plot has six components: (a) pseudo-chromosome number; (b) gene density; (c) density of TEs; (d) single-nucleotide polymorphism (SNP) density; (e) density of GC content; and (f) genomic synteny.

To reduce the heterozygosity in wild black wolfberry (*L. ruthenicum*), pollen culture was used to obtain haploid material from *L. ruthenicum* accession ‘Heiguo’ (Fig. S1a), which was used for genome assembly and annotation. Karyotype analysis confirmed the haploidy of ‘Heiguo’, with *n* = 12 chromosomes (Fig. S1b). Subsequently, PacBio sequencing was used to obtain 71.05 Gb of HiFi data, which covered ~31 × of the genome. The contig N50 length of the HiFi data was 14.73 kb, with an average length of 14.16 kb. In addition, 199.76 Gb of Hi-C data were generated, which covered ~88 × of the genome. Finally, contig assembly and chromosome anchoring were combined to produce an assembly of 2272 Mb comprising 12 chromosomes, with a contig N50 of 92.64 Mb and a scaffold N50 of 188.11 Mb (Table 1). The anchored sequence length on the chromosomes was 2214.45 Mb, with a Hi-C anchoring rate of 97.46% (Fig. 1a). Three methods were used to evaluate the quality of the genome. First, Benchmarking Universal Single-Copy Orthologues (BUSCO) indicated that 99.10% (1600/1614) of core genes were completely captured in the genome assembly. Second, the long terminal repeat (LTR) assembly index (LAI) value was 12.27. Finally, the QV score was 66.39. In total, 1854 Mb (81.60%) of repeat sequences were annotated in the genome. Most of these repeats were LTR retrotransposons, accounting for 66.66% of repeat sequences (Fig. 1b; Table S1). Among the transposable element (TE) superfamilies, the amplification of LTR/Gypsy elements was found to be a major driving force for the inflation of the *L. ruthenicum* genome, with Copia, Gypsy, and unknown types of LTR-RT accounting for 2.40%, 37.49%, and 26.77% of the genome, respectively. A total of 38 993 gene models were predicted using a combination of *ab initio* prediction, homology-based prediction, and transcriptomic-assisted prediction, with 35 294 non-redundant gene models (90.51%) being annotated by at least one of the five databases (Gene Ontology (GO), Kyoto Encyclopedia of Genes and Genomes (KEGG), COG, Nr, and Swiss–Prot). Using the online plantiSMASH tool (http://plantismash.secondarymetabolites.org/about.html), 40 gene clusters were identified (e.g. terpene, saccharide, alkaloid, polyketide, etc.), distributed across 12 chromosomes (Table S2). These results indicate that the *L. ruthenicum* genome assembly was of high quality and largely improved compared with the published draft genome [48].

### Comparative genomics and genome evolution of *L. ruthenicum*

**Figure 2:**
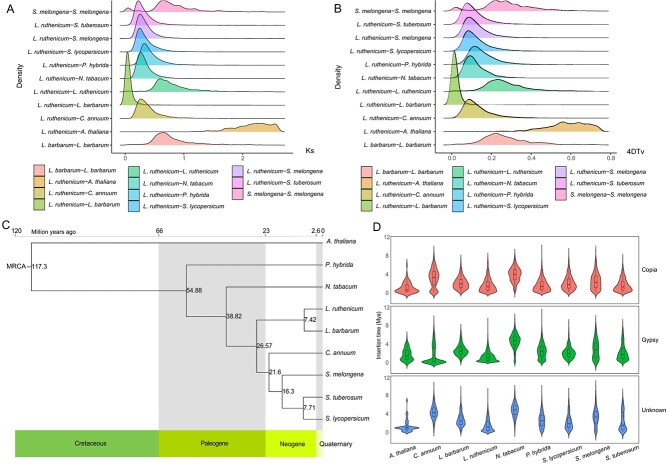
Genome evolution and WGD in *L. ruthenicum* and eight other species. (A) Distribution of Ks values of 11 comparisons (*L. barbarum* − *L. barbarum*, *L. ruthenicum* – *A. thaliana*, *L. ruthenicum* – *C. annuum*, *L. ruthenicum* − *L. barbarum*, *L. ruthenicum* − *L. ruthenicum*, *L. ruthenicum* – *N. tabacum*, *L. ruthenicum* – *P. hybrida*, *L. ruthenicum* – *S. lycopersicum*, *L. ruthenicum* – *S. melongena*, *L. ruthenicum* – *S. tuberosum*, *S. melongena* − *S. melongena*). (B) Distribution of 4DTv values of 11 comparisons (*L. barbarum* − *L. barbarum*, *L. ruthenicum* − *A. thaliana*, *L. ruthenicum* − *C. annuum*, *L. ruthenicum* − *L. barbarum*, *L. ruthenicum* − *L. ruthenicum*, *L. ruthenicum* − *N. tabacum*, *L. ruthenicum* − *P. hybrida*, *L. ruthenicum* − *S. lycopersicum*, *L. ruthenicum* − *S. melongena*, *L. ruthenicum* − *S. tuberosum*, *S. melongena* − *S. melongena*). (C) Phylogenetic trees and divergence times of the nine species examined. (D) Different LTR-RT insertion times of the nine species examined.

Whole-genome duplication (WGD) is a common phenomenon in plant evolution, driving the complexity and stability of plant genome regulatory networks. It directly increases the regulatory units of the network and provides a source for the generation of new genes and subfunctionalization, having an important role in enhancing plant diversity and environmental adaptability. To explore the evolutionary history of WGD events in *L. ruthenicum*, Ks and 4-fold degenerate third-codon transversion (4DTv) values were calculated based on homologous gene pairs in *L. ruthenicum* and eight other species (Fig. 2a, b). The Ks and 4DTv plots exhibited similar density patterns. The distribution of Ks values for paralogous genes in *L. ruthenicum* showed a peak at ~0.65 (4DTv ~0.24–0.27), indicating that *L. ruthenicum* had experienced a WGD event. Genome comparisons revealed that *L. ruthenicum* and *L. barbarum* exhibited the lowest Ks and 4DTv peak, indicating a closer relationship and more similar homologous gene differentiation. This comparison was followed by *L. ruthenicum* and *Nicotiana tabacum*, *Solanum tuberosum*, *Capsicum annuum*, and *Solanum melongena*, followed by *Petunia hybrida*, and finally *Arabidopsis thaliana*, which is consistent with the evolutionary relationships of their genomes (Fig. 2a, b). The Ks peaks between *L. ruthenicum* and other Solanaceae species were between 0.4 and 0.5, suggesting that these species share a WGD event, in accordance with a previous report of a shared WGD event in Solanaceae [48]. Subsequently, to clarify the phylogenetic position of *L. ruthenicum*, comparative genomics analysis was performed using the genomes of eight Solanaceae species (*N. tabacum, S. tuberosum, C. annuum, S. melongena, P. hybrida, Solanum lycopersicum, L. ruthenicum*, and *L. barbarum*) and the outgroup *A. thaliana*. A phylogenetic tree was constructed based on 136 single-copy genes in *L. ruthenicum* and these eight other species (Fig. 2c), which showed that *L. ruthenicum* was most closely related to *L. barbarum*, with a divergence from *L. barbarum* at ~7.42 million years ago (Mya), which was after the divergence of the common ancestor of *L. ruthenicum* and *C. annuum* (26.57 Mya).

Given that LTR-RTs were predominately detected, the category and insertion time of different lineages of LTR-RTs in *L. ruthenicum* and the genomes of the eight other species were determined. In *L. ruthenicum*, the Copia, Gypsy, and unknown types accounted for 9.24%, 63.84%, and 26.91% of LTR-RTs (Fig. S2). Recent bursts were found of Copia (~1.57 Mya) and Gypsy (~1.18 Mya) in *L. ruthenicum*, which were similar to that in *P. hybrida* for Copia (~1.58 Mya), but later than Copia in *C. annuum* (~3.09 Mya), *N. tabacum* (~3.79 Mya), and Gypsy in *N. tabacum* (~4.53 Mya) (Fig. 2d).

### Gene family analysis of Solanaceae species

**Figure 3:**
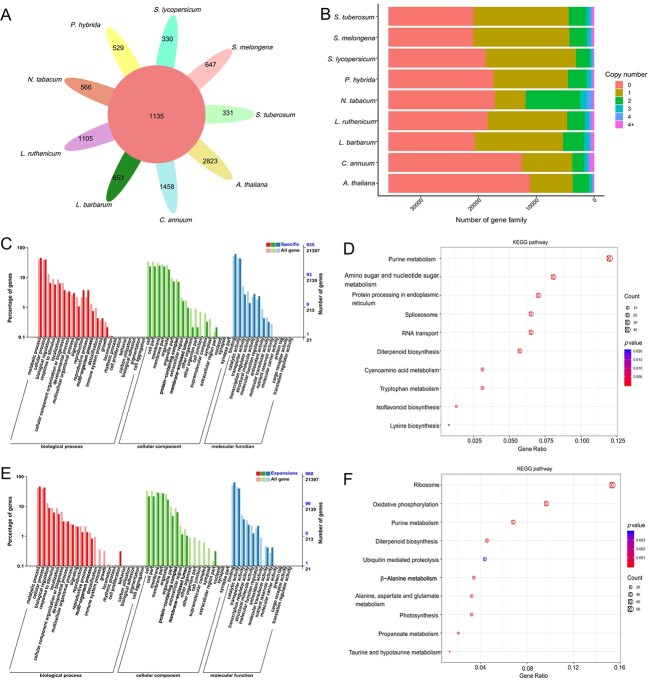
Gene family analysis of the nine species involved in this study. (A) Venn diagram of shared and unique gene families across the nine species. (B) Copy number distribution of gene families in the nine species. GO (C) and KEGG (D) enrichment analyses of the specific gene families in *L. ruthenicum*. GO (E) and KEGG (F) enrichment analysis of the expanded gene families in *L. ruthenicum*.

The gene models of the nine species (Solanaceae species and *Arabidopsis*), containing a total of 291 730 genes, were clustered into 35 600 orthogroups, which included 8442 species-specific orthogroups. In total, 1135 gene families were shared among the nine species, with 1105 gene families specific to *L. ruthenicum* (Fig. 3a; Fig. S3), which was the second highest number of specific gene families, the highest being 2823 in *A. thaliana*. *Lycium ruthenicum* contained 17 121 zero-copy gene families, the highest proportion in the genome (48.1%), which is comparable to that in tomato (47.2%); followed by two-copy gene families, accounting for 38.80%, which was similar to that in *P. hybrida* (36.20%) (Fig. 3b). GO enrichment analysis revealed that the specific gene families in *L. ruthenicum* were mainly involved in metabolic biological processes, membrane-related cellular components, and binding molecular functions (Fig. 3c). KEGG enrichment analysis showed that these specific gene families were mainly associated with metabolism pathways, such as purine, amino sugar and nucleotide sugar, cyanoamino acid, and tryptophan (Fig. 3d).

In the genome of *L. ruthenicum*, 306 gene families showed significant expansion (*P* < 0.05), while 197 exhibited significant contraction (Fig. S4). The GO enrichment of expanded families resulted in a similar scenario to the GO enrichment analysis of specific genes in *L. ruthenicum* (Fig. 3e). In addition, KEGG pathway analysis showed that these expanded gene families were mainly related to environmental stress response, such as ribosome (*P* = 4.91E-21), oxidative phosphorylation (*P* = 2.94E-16), and purine metabolism (*P* = 1.13E-8) (Fig. 3f), indicating that the extreme environmental adaptation of *L. ruthenicum*, such as drought, salinity, and high ultraviolet (UV) radiation adaptability, may benefit from the expansion of oxidative stress-related genes [49–52]. Positive selection analysis identified 210 significantly positive selected genes (Table S3), some of which were enriched in the base excision repair pathway (evm.TU.Chr02.3333/evm.TU.Chr06.1683/evm.TU.Chr06.2972) and homologous recombination pathway (evm.TU.Chr02.3333/evm.TU.Chr03.2887/evm.TU.Chr06.570) (Fig. S5). *Lycium ruthenicum* grows in high-altitude barren areas and, therefore, must withstand intense UV radiation, which inevitably causes DNA damage [53, 54]. We speculate that, as a result, *L. ruthenicum* has evolved DNA repair systems to adapt to strong UV radiation.

### Chromosome synteny between *L. ruthenicum* and *L. barbarum*

**Figure 4:**
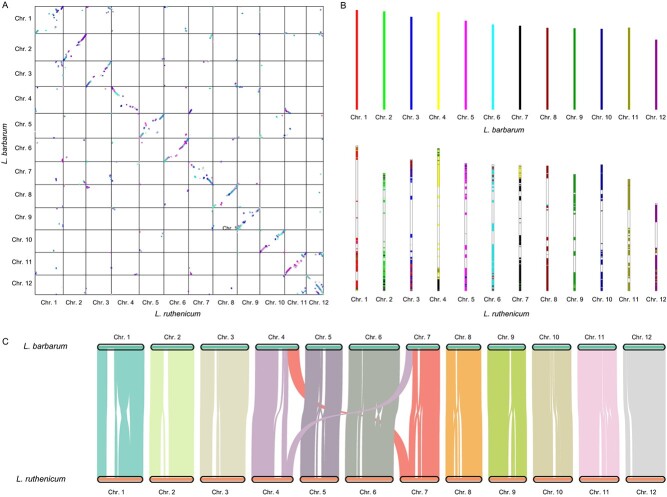
Genome synteny analysis. (A) Syntenic analysis of *L. barbarum* and *L. ruthenicum* genomes. (B) Chromosome rearrangements between *L. barbarum* and *L. ruthenicum*. (C) Chromosome alignment between *L. barbarum* and *L. ruthenicum*.

Aligning the *L. ruthenicum* and *L. barbarum* genomes using MCScanX and BLASTP provided information on genome collinearity between the two genomes (Fig. 4a–c), yielding a total of 531 syntenic blocks containing 18 890 genes. These genes accounted for 48.44% of the genes in the *L. ruthenicum* genome and 48.16% of the genes in the *L. barbarum* genome, averaging 35.57 genes per block. The collinear blocks were predominantly located in the terminal regions of the chromosomes, suggesting unequal evolution among different chromosome segments (Fig. 4b). The most collinear blocks were found on chromosome 1 of *L. ruthenicum,* with 74 blocks, whereas the fewest were found on chromosome 05, with 29 blocks (Fig. 4b). This indicates that the genome assembly of *L. ruthenicum* shows general continuity and is consistent with their close phylogenetic relationship as members of the *Lycium* clade. Further analysis of the chromosomes where collinear blocks were located revealed fusion or fragment events between the terminal regions of chromosome 04 in *L. ruthenicum* and chromosome 07 in *L. barbarum*, as well as between the terminal regions of chromosome 07 in *L. ruthenicum* and chromosome 04 in *L. barbarum* (Fig. 4c).

To further investigate chromosomal variations between *L. ruthenicum* and *L. barbarum*, structural variation (SV) detection was performed for variations >50 bp. In total, 63 414 SVs were identified, including insertions (6075, 9.58%), inversions (10 400, 16.40%), duplications (32 160, 50.71%), and translocations (14 779, 23.31%); overall, 2194 inserted, 7163 inverted, 694 duplicated, and 1197 translocated genes were annotated (Table S4), which could explain the phenotypic differences between *L. ruthenicum* and *L. barbarum*. These findings suggest that, despite extensive chromosomal rearrangements, *L. ruthenicum* and *L. barbarum* still share chromosomes from their common ancestor. Overall, these findings shed new light on the evolution of *Lycium* chromosomes.

### Characterization of genes involved in anthocyanin biosynthesis

Unlike *L. barbarum*, *L. ruthenicum* is rich in anthocyanins, which have important biological activity. To investigate the reasons for this difference, genomic, transcriptomic, and metabolomic methods were combined to determine the genes regulating anthocyanin biosynthesis during fruit development in *L. ruthenicum*. In total, 87 candidate genes encoding enzymes involved in anthocyanin biosynthesis were identified, including 45 encoded constitutive enzymes; 29 encoded TFs; and 13 encoded transporters (Table S5). Expression of 63 anthocyanin biosynthesis-related genes [fragments per kilobase of transcript per million mapped reads (FPKM) >1] was found in fruits (Table S5). Furthermore, 28, 21, 16, and 24 differentially expressed genes (DEGs) showed significantly higher expression in ‘Heiguo’ compared with four other *L. ruthenicum* accessions: ‘HZ-13-01’, ‘Zh-13-0802’, ‘QH-13-0806’, and ‘Zhuxi07’, respectively (Table S6). The inferred increase in transcript abundance might contribute to the increased anthocyanin accumulation in ‘Heiguo’.

**Figure 5:**
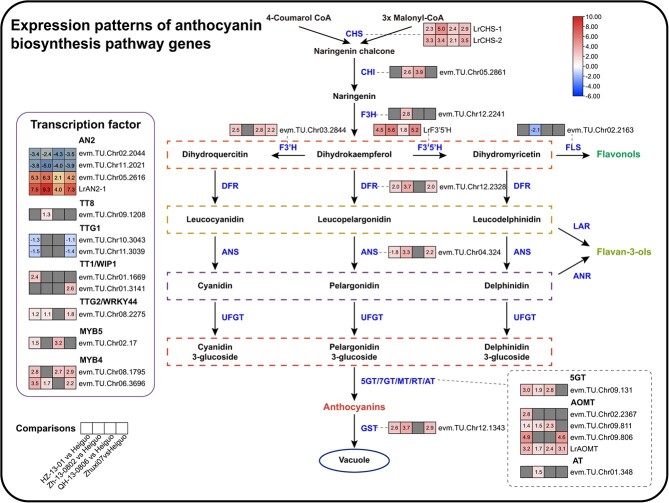
Expression patterns of anthocyanin biosynthesis pathway genes and TFs. The enzymes encoded by the related DEGs are located next to the structural genes in the anthocyanin biosynthesis pathway. The four adjacent square heat maps from left to right represent the corresponding DEGs in comparison groups ‘HZ-13-01’ versus ‘Heiguo’, ‘Zh-13-0802’ versus ‘Heiguo’, ‘QH-13-0806’ versus ‘Heiguo’, and ‘Zhuxi07’ versus ‘Heiguo’, respectively.

Among these significantly upregulated genes, two AN2-type TFs (*evm.TU.Chr05.2616* and *evm.TU.Chr05.2618* (*LrAN2–1*)) were highly upregulated in all four comparison groups. In ‘Heiguo’, their expression levels were 35.43 and 161.64 times higher than in ‘HZ-13-01’, 77.05 and 631.49 times higher than in ‘Zh-13-0802’, 4.27 and 15.53 times higher than in ‘QH-13-0806’, and 18.59 and 161.13 times higher than that in ‘Zhuxi07’, respectively. The F3’5’H gene (*evm.TU.Chr06.177* (*LrF3’5’H*)) and the two CHS genes (*evm.TU.Chr05.295*(*LrCHS1*) and *evm.TU.Chr09.488*(*LrCHS2*)) were significantly upregulated in ‘Heiguo’ in all four comparisons (‘HZ-13-01′ versus ‘Heiguo’, ‘Zh-13-0802′ versus ‘Heiguo’, ‘QH-13-0806′ versus ‘Heiguo’, and ‘Zhuxi07’ versus ‘Heiguo’; Fig. 5). Furthermore, one AOMT gene, *evm.TU.Chr09.809* (*LrAOMT*), was also differentially expressed in the four comparisons. Pearson correlation analysis of gene expression levels and anthocyanin-related metabolites revealed that *LrAN2.1*, *LrCHS1*, and *LrCHS2* were significantly correlated with 12 anthocyanin-related metabolites (r > 0.9 or < −0.9, *P* < 0.05) (Fig. S6; Table S7). These results indicated that these genes have a potentially crucial role in anthocyanin biosynthesis or its regulation in the fruit of *L. ruthenicum*. Further exploration of the specifically expanded gene families that might be involved in anthocyanin biosynthesis showed that the 86 genes belonged to 65 orthogroups, 17 of which had more than two copies in the *L. ruthenicum* genome. However, none of these orthogroups were found to have rapidly expanded in the *L. ruthenicum* genome compared with the eight related species.

### Functional characterization of anthocyanin biosynthesis pathway genes

To further validate the function of anthocyanin biosynthesis pathway genes and potential regulatory TFs identified in the *L. ruthenicum* genome, transient transformation was conducted in *L. barbarum* leaves using genes based on the expression profiles in Fig. 5 and the correlation between gene expression and anthocyanin-related metabolites in Fig. S6, including two CHS-type genes called *LrCHS1* (evm.TU.Chr05.295) and *LrCHS2* (evm.TU.Chr09.488), as well as *LrAOMT* (evm.TU.Chr09.809), *LrF3’5’H* (evm.TU.Chr06.177), and *LrAN2.1* (evm.TU.Chr05.2618). The target genes showed significantly higher expression levels compared with the CK (GFP) in overexpression plants (Fig. S7). Anthocyanin metabolite detection showed significant upregulation of delphinidin 3-O-galactoside chloride, peonidin-3-O-glucoside chloride, cyanidin O-syringic acid, cyanidin 3-glucoside, and procyanidin B1 in LrAOMT-overexpressing (OE) plants, whereas cyanidin 3-rutinoside (keracyanin chloride) was significantly downregulated. Overexpression of *LrCHS1* significantly increased the accumulation of procyanidin B1 and pelargonidin chloride; and overexpression of *LrCHS2* significantly or extremely significantly promoted the procyanidin B1, procyanidin B2, and cyanidin 3-O-glucoside contents. In *LrF3’5’H*-OE plants, only the expression of procyanidin B1 was significantly upregulated, whereas apigeninidin chloride, pelargonidin 3-O-β-D-glucoside (callistephin chloride), and malvin (chloride) were significantly or extremely significantly inhibited. Overexpression of the TF *LrAN2.1* caused significant downregulation of pelargonidin 3-O-β-D-glucoside.

To further clarify the effects of these overexpressed genes on other anthocyanin biosynthesis pathway genes, RNA-sequencing (RNA-seq) and DEGs analysis were conducted on the transiently transformed samples (Fig. S8). The results showed that the target genes were significantly upregulated, in line with the quantitative (q) PCR results. In addition, overexpression of *LrCHS2* caused downregulation of *Lr4CL* (evm.TU.Chr03.2248), *LrMYB4* (evm.TU.Chr08.1795), and *LrF3H* (evm.TU.Chr12.2241); overexpression of *LrAOMT* caused upregulation of *LrAHA6/8* (evm.TU.Chr04.2987) and *LrGSTF8* (evm.TU.Chr09.746), but downregulation of *LrTT1* (evm.TU.Chr01.1669), *LrCHI* (evm.TU.Chr05.2861), *LrTTG2* (evm.TU.Chr08.2275), and *LrDFR* (evm.TU.Chr12.2328); and overexpression of *LrF3’5’H* caused upregulation of *LrCHS1* (evm.TU.Chr05.295), *LrMYB4* (evm.TU.Chr08.1795), and *LrCHS2* (evm.TU.Chr09.488), but downregulation of *LrAN2.1* (evm.TU.Chr05.2618) (Table S8). These results indicated that anthocyanin biosynthesis in *L. ruthenicum* is influenced by multiple structural genes at different levels and is also regulated by TFs.

## Discussion

*Lycium ruthenicum* Murr. is an important edible and medicinal plant in traditional Chinese medicine. It is enriched with anthocyanin, flavonoids, triterpenoids, polysaccharides, amino acids, alkaloids, and other recognized biologically active substances. This study assembled a high-quality genome of *L. ruthenicum* and used it as a base to investigate the anthocyanin biosynthesis pathway. This complete genome sequence provides a foundation for functional genome research and phylogeny in *Lycium* species and perhaps other medicinal plants.

### The chromosome-level *L. ruthenicum* genome provides a benchmark for genetic and functional genomic research

The *L. ruthenicum* genome is characterized by high heterozygosity, making it challenging to assemble [48]. In this study, we developed haploid *L. ruthenicum*, and combined PacBio HiFi sequencing and high-throughput Hi-C technologies to generate a chromosome-level high-quality genome, with a scaffold N50 of 188.11 Mb and a contig N50 of 92.64 Mb, showing a higher continuity than the genome of *L. barbarum* (contig N50 = 50.55 Mb) [46]. BUSCO analysis found that 99.10% of the core conserved genes in the assembled genome were captured. Moreover, LAI and QV values of 12.37 and 66.39, respectively, highlight the greater integrity and higher quality of this *L. ruthenicum* genome compared with its published draft genome, which harbored a contig N50 of 16.14 kb and a scaffold N50 of 155.39 kb [48], and those recently published for other medicinal plants [55, 56, 114]. This *L. ruthenicum* reference genome provides a new perspective for understanding gene structure, composition, function, gene regulation, and species evolution at the molecular level. It is also of value for improving the agronomic and medicinal characters of *L. ruthenicum* via molecular breeding.

We also used the Illumina sequencing platform to sequence the RNA of other *L. ruthenicum* accessions with black fruits and found a relatively low properly mapping rate (<40%) that might limit its application to such accessions; thus, a more representative assembly of *L. ruthenicum* might be required because of its highly differentiated genome. Recently, the telomere to telomere (T2T) genomes of *Rhodomyrtus tomentosa* [57], *Vitis vinifera* [58], *Actinidia chinensis* [59], and *Panax ginseng* [60], among others, were successfully assembled and reported. We checked the telomeres in our assembly using the seven-base telomeric repeat (CCCTAAA at the 5′ end or TTTAGGG at the 3′ end) as a sequence query [61]. In addition, five candidate centromeric tandem repeats were used to detect candidate centromeric homologous sequences and locations [62]. Regrettably, no BLAST hit was identified with an E threshold of 1e-5 for centromeres and telomeres in our assembly. To achieve the T2T assembly level, the ultralong reads generated by Nanopore could be combined to fill the gaps in telomeres and centromeres, which will be a focus for future work.

### Genome differentiation of *L. ruthenicum* and *L. barbarum*

The genome assembled in this study for *L. ruthenicum* was 2.21 Gb in size, which is ~0.5 Gb (22.13%) larger than the recently reported genome size of *L. barbarum* (1.7 Gb) [46], whereas the predicted gene counts differed by <1% (38 993 vs 39 224). The *L. ruthenicum* genome was annotated with a total of 1.85 Gb repetitive sequences, whereas that of *L. barbarum* had 0.82 Gb of annotated repetitive sequences, suggesting that repetitive sequences, mainly Gypsy-type LTR-RTs, have undergone expansions leading to the increased genome size of *L. ruthenicum*. Additionally, repetitive sequences were concentrated near or in the centromere regions, which are known to have a faster evolutionary rate [63]. This could explain why collinear segments of the *L. ruthenicum* and *L. barbarum* genomes are located at the chromosome arm (Fig. 4). However, further support from genome sequences of different *Lycium* species is still needed to fully understand the diversity and driving forces of repetitive sequence differentiation. Variant detection analysis revealed numerous SVs between the *L. ruthenicum* and *L. barbarum* genomes, including a total of 14 565 inserted genes, 7163 inverted genes, and 15 150 deleted genes (Table S4), suggesting that large-scale SVs have led to functional divergence in genes. These genes were enriched in pathways related to environmental adaptation, such as the oxidative phosphorylation pathway (ko00190, *P* < .0001) and the photosynthesis pathway (ko00195, *P* < .0001). Interestingly, the expanded genes and positively selected genes in the L*. ruthenicum* genome also showed enrichment in environmental adaptation, particularly in UV and DNA damage repair pathways (Fig. 3c–f), which aligns with the abiotic or biotic stresses present in the natural habitat of *L. ruthenicum*, such as drought, high salinity, and strong UV exposure. Positive selection acting on DNA damage and repair-related genes has also been observed in other species exposed to intense UV radiation, such as *Hordeum vulgare* L. var. *nudum* (Tibetan hulless barley; [64]), *Salix brachista* (cushion willow; [65]), *Crucihimalaya himalaica* [117], and *Populus cathayana* [66]. These results imply that *L. ruthenicum* has undergone adaptive evolution in response to its environment, providing new insights into the adaptive mechanisms of plants in response to strong UV and drought environments. Using plantiSMAH to predict the metabolic synthesis clusters of *L. barbarum* and *L. ruthenicum* genomes (Table S2), we found that *L. ruthenicum* has undergone differentiation in the biosynthesis of certain secondary metabolites, such as polyketide-alkaloid and saccharide-polyketide, of which alkaloid has been studied for its association with adaptive responses to environmental stress [67, 68]. In summary, the genome sequence and gene functions of *L. ruthenicum* have undergone extensive differentiation compared with those of *L. barbarum*.

**Figure 6:**
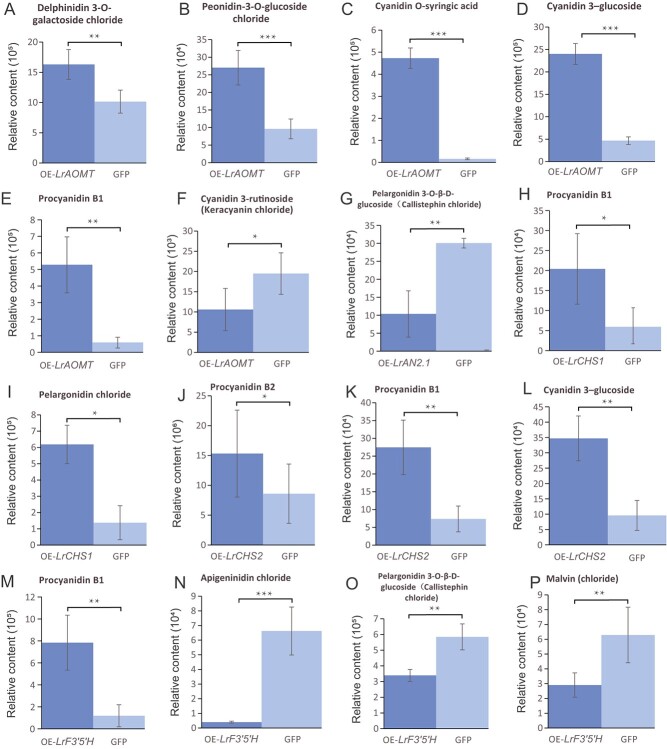
Differential expression of anthocyanin-related metabolites in overexpression plants. (A–F) *LrAOMT*-OE plants; (G) OE-*LrAN2.1* plants; (H, I) OE-*LrCHS1* plants; (J–L) OE-*LrCHS2* plants; (M–P) OE-*LrF3’5’H* plants. Student’s *t*-test was used for significance comparisons: ^*^P < 0.05; ^**^P < 0.01; ^***^P < 0.001.

### Complex regulatory mechanisms of anthocyanidin biosynthesis in *L. ruthenicum*

The biosynthesis pathway of anthocyanins has been well revealed in different species mainly using homologous cloning or transcriptional expression [14, 69, 70]. In the present study, we assembled a *L. ruthenicum* variety rich in anthocyanins, and identified 86 homologous genes and potential regulators in the anthocyanin biosynthesis pathway, and profiled their expression patterns (Tables S4 and S5). Combined with differential expression analysis, we screened out 16 differentially expressed structural genes and 12 differentially expressed TFs for anthocyanin biosynthesis (Fig. 5). In addition, we found three genes in the anthocyanin synthesis pathway (evm.TU.Chr05.2618, evm.TU.Chr09.488, and evm.TU.Chr05.295) that showed significant correlations (positive or negative) with the related metabolites in *L. ruthenicum* (Fig. S6), indicating that different biosynthesis and regulatory mechanisms might exist in this species. We further screened out four structural genes with higher expression levels and stable differential expression and one MYB-type TF, and confirmed their functions through transient transformation experiments (Fig. 6). In general, the selected structural genes *LrAOMT*, *LrCHS1*, *LrCHS2*, and *LrF3’5’H* were found to be involved in promoting anthocyanin biosynthesis, which is consistent with published reports [71–74]. MYB TFs regulate anthocyanin biosynthesis by binding to the promoter region of structural genes directly or via the MBW complex [28]. Our study found that *LrAN2.1* reduced the pelargonidin 3-O-β-D-glucoside content (Fig. 6), indicating its role as a negative regulator of anthocyanin biosynthesis. In addition, *LrMYB4* was found to be up- and downregulated in OE-*LrCHS2* and OE-*LrF3’5’H* plants, respectively, suggesting that *LrMYB4* might be affected by multiple carotenoid synthesis pathway genes [75]. However, whether there are interactions between structural genes and this MYB TF requires further verification, such as using yeast one-hybrid (Y1H) or dual-luciferase assays [35, 76]. Furthermore, by sequencing the transcriptome of plants overexpressing certain genes, we found that, in OE-*LrCHS2*, OE-*LrAOMT*, and OE-*LrF3’5’H* plants, other structural genes and TFs in the anthocyanin biosynthesis pathway were significantly upregulated or downregulated, indicating that these genes affect multiple anthocyanin biosynthesis pathway genes. Other genes that have not yet been validated in transient transformation experiments, such as *LrF3’H* (evm.TU.Chr03.2844) and *LrCHI* (evm.TU.Chr05.2861), have been reported to participate in anthocyanin biosynthesis [77, 78]. However, *LrF3’H* (evm.TU.Chr03.2844) was only differentially expressed in the comparison groups of ‘Heiguo’ versus ‘Zh-13-0802′ and ‘Heiguo’ versus ‘QH-13-0806′, whereas *LrCHI* (evm.TU.Chr05.2861) was differentially expressed in the comparison groups of ‘Heiguo’ versus ‘HZ-13-01′, ‘Heiguo’ versus ‘QH-13-0806′, and ‘Heiguo’ versus ‘Zhuxi07’, suggesting that these genes also have a role in anthocyanin biosynthesis in *L. ruthenicum*, but that there are diverse mechanisms in different accessions, further highlighting the complexity of anthocyanin biosynthesis and regulation in L*. ruthenicum*.

## Conclusions

This study reported a high-quality chromosome-scale genome of *L. ruthenicum,* and comparative genomic analyses revealed sequence and potential functional differentiation between *L. ruthenicum* and *L. barbarum*, providing new insights into the genomic relationship between these two species. Using multiomics data, we identified genome-wide anthocyanin biosynthesis genes and validated the function of some key DEGs, such as *LrCHS, LrAOMT*, *LrF3’5’H*, and *LrAN2.1*, laying a foundation for subsequent interactions between structural gene and TFs in the regulation of anthocyanin biosynthesis. This genome assembly and anthocyanin biosynthesis genes revealed will be important resources for accelerating functional and evolutionary genomic research, as well as the molecular breeding or genome editing of *L. ruthenicum* to enhance anthocyanin production to fully expand and support the use of this important health food and traditional Chinese medicine.

## Materials and methods

### Plant material preparation, sampling, and karyotype identification

The *L. ruthenicum* accessions were acquired from Chinese National Wolfberry Engineering Research Center, and haploid plants were derived from pollen culture [79]. At seedling stage, the young leaves of haploid plants were sampled for next-generation sequencing (DNA sequencing) and PacBio HiFi sequencing library construction (Adsen Biotechnology Co., Ltd., Urumchi, China). The fresh young leaves were collected and immediately processed for library construction. Mature fruits of 5-year old ‘Heiguo’, ‘HZ-13-01’, ‘Zh-13-0802’, ‘QH-13-0806’, and ‘Zhuxi07’ plants were harvested with three biological repetitions, followed by freezing in liquid nitrogen and storage (−80°C) for RNA-seq, metabolome, and qRT-PCR experiments. The leaves of 35-day seedlings of ‘Ningqi NO.1’ were used for transient overexpression experiments. Karyotype analysis was performed according to the method of Lan et al. [80] by using root tip tissue of the haploid ‘Heiguo’.

### Library constructions and high-throughput sequencing

Genomic DNA from young leaves (~10 g) was isolated and ~10 μg DNA was sheared into ~20 kb by Megaruptor 2 (Diagenode, Denville, USA), and an SMRTbell library was constructed according to Pacific Biosciences guidelines (Pacific Biosciences, CA, USA). Then, size selection was performed (~3-μg fragments) by using Sage ELF (Sage Science, Beverly, MA, USA) to collect SMRTbells of ~20 kb. Long-read HiFi sequencing was performed on the PacBio Sequel II (Pacific Biosciences, CA, USA). To prepare Hi-C library, fresh leaves from the same plants used for HiFi sequencing were immediately crossed using formaldehyde, digested with enzyme Hind III, and followed by end-repairing by a biotin-modified base. Circular DNA was continuously generated and fragmented into 300- to 700-bp fragments. Finally, the fragments were enriched by biotin beads. After library quantification by Qbit 2.0 (Life Technologies, Carlsbad, CA, USA), the Hi-C library was sequenced (PE150) on Illumina Novaseq 6000 (Illumina, San Diego, CA, USA).

### Genome assembly, annotation, and evaluation

The subreads generated by PacBio Sequel II were assembled into contigs using Hifiasm (0.16.1) under HiFi + Hi-C mode (parameters: -k 51 -a 4 -m 10 000 000 -x 0.8 -y 0.2) [81]. The hicup_truncater module from hicup_v0.5.9 was used to remove sequences containing restriction enzyme cut sites from the reads before aligning them to the contig genome. Uniquely mapped data (quality value >20) were used for subsequent chromosome scaffolding. The genome sequence was partitioned, sorted, and oriented using the SALSA2 software [82] under option ‘-m no -e AGCTT’. The created assembly was further manually error corrected [83]. The genome was renamed and the final sequence generated using agptools (https://github.com/WarrenLab/agptools). The quality of this genome was evaluated based on the alignments of 1614 conserved genes of BUSCO V5 (embryophyta_odb10 database) [22]. The LAI v2.9.9 [84] and QV [85] values were also adopted for genome quality assessment.

Extensive *de novo* TE Annotator (EDTA) software v2.0.1 [86] was adopted to generate a non-redundant repeat sequence database with optional parameters ‘--sensitive 1 --evaluate 1 --anno 1’. RepeatMasker [87] was then used to annotate and mask repetitive sequences in the genome using this database. The GeMoMaPipeline module was utilized for gene prediction [88]. Using the principle of homology prediction and referencing the published draft *L. barbarum* genome [48], gene prediction was carried out under parameters ‘p=true o=true AnnotationFinalizer.r=SIMPLE’. In addition to homology-based gene structure prediction, Braker2 software v2.1.6 was used for *ab initio* prediction with the parameters ‘--epmode --softmasking --prot_seq=odb10_plants_fasta’ of EP mode (ProtHint+ GeneMark-EP + AUGUSTUS) [89], which involved training models and predicting genes using plant protein sequences from the OrthoDB database. By considering evidence support, a reliable set of gene predictions (FULLSUPPORT) was obtained as the final prediction dataset. Based on RNA sequence assembly of EST/Unigenes (PRJNA640228), TransDecoder (http://transdecoder.github.io/) was used to predict gene models. EVidenceModeler was utilized to merge the gene data set from Braker2 and GeMoMa into a non-redundant and complete gene prediction result [90] (options: --segmentSize 100 000 --overlapSize 10 000). For functional annotation, the protein sequences translated according to the final gene model set were mapped against five databases (UniProtKB/Swiss–Prot, Nr, GO, COG, and KEGG) using BLASTP with a sequence identity >50% and an E-value cut-off of 1E-5.

### Synteny analysis and structural variation calling

The genome assemblies of *L. barbarum* [46] were aligned to this *L. ruthenicum* genome using minimap2 [115] with the parameters ‘-t 20 -ax asm5 –eqx’ and alignment filtering (options: ‘-1 -i 90 -l 500’), followed by SV calling [91]. The genes harbored by SVs were subjected to GO and KEGG analyses.

### Gene family and phylogenetic-related analyses

Proteins from each gene from the annotation files of nine species (*A. thaliana, L. ruthenicum, S. tuberosum, L. barbarum, S. melongena, C. annuum, P. hybrida, N. tabacum*, and *S. lycopersicum*) were isolated for family clustering by using OrthoFinder v2.4.0 (diamond, e = 0.001) [92], followed by gene family annotation based on the PANTHER V15 database [93]. To reveal phylogenetic relationships among these species, single-copy ortholog protein sequences were used to construct a phylogenetic tree. The proteins were aligned by using MAFFT v7.205 (options: --localpair --maxiterate 1000) [94] for further phylogenetic tree construction by using IQ-TREE [95]. ModelFinder [96] identified the best model as JTT + F + I + G4, which was then used to construct a maximum likelihood phylogenetic tree (bootstrap = 1000). The CAFE v4.2 software package [97] was selected to mine gene families under significant expansion or contraction (family-wide *P-*values <0.05 and viterbi *P-*values <0.05). We utilized the CodeML module (F3x4 model of codon frequencies) within PAML [98] for positive selection analysis.

### Whole-genome duplication and the insert time of LTR analyses

In the present study, both the Ks and 4DTv were used to determinate WGD using wgd (v1.1.1) [99] and calculate_4DTV_correction.pl (https://github.com/JinfengChen/Scripts). A combination of LTR_finder (v1.07) and LTRharvest (v1.5.10) was used to call the full-length LRT-RTs (fl-LTR-RTs) [100, 101]. LTR_retriever was used to prepare fl-LTR-RTs and non-redundant LTR library [102], followed by extraction of LRT flanking sequences and mapping using MAFFT (v7.205) under parameters —localpair —maxiterate 1000 [94]. Finally, the distance was estimated in EMBOSS by selecting the Kimura model [103].

### RNA-seq analysis

The mature fruits of ‘HZ-13-01’, ‘Zh-13-0802’, ‘QH-13-0806’, and ‘Zhuxi07’ accessions at S5 stage (Fig. S9) [104] and transient overexpression leaves of NQ No.1 were sampled with three biological replicates for RNA-seq. RNA-seq libraries were prepared and sequenced following Illumina’s protocols (Illumina, San Diego, CA, USA). Raw reads underwent data quality control according to Zhao et al. [104] to harvest clean data. The clean data were mapped to the *L. ruthenicum* genome using STAR [105] under default parameters, followed by transcript assembly using StringTie [106]. The FPKM was used for transcript expression quantification [107]. All the genes with FPKM value>1 were retained for the following analysis. Finally, DEGs were determined using TBtools [113] with *P* < 0.01 and fold change ≥1.5. GO and KEGG were conducted as enrichment analyses [108, 119].

### Anthocyanin content determination and data analysis

Anthocyanin was extracted from three independent fruits of the five *L. ruthenicum* accessions and analyzed using an UPLC-ESI-MS/MS system (Applied Biosystems, CA, USA) [109]. For overexpression materials, the fresh samples were crushed (Restch) with zirconia beads for 1.5 min at 30 Hz. The powder (10 mg) was extracted at 4°C (1.0 ml of 70% aqueous methanol) for 24 h. After centrifugation for 10 min at 10 000 ***g***, the extracts were filtered (SCAA-104, 0.22-mm pore size, ANPEL), and LC–MS analysis was then performed following the method of Chen et al. [110]. All the procedures were done on ice in the dark. Differential expressed metabolites (DEM) were identified by the Student’s *t-*test. The correlation between DEMs and DEGs was calculated using Pearson’s correlation coefficient (Hmisc package R).

### Identification and validation of homologous genes in the anthocyanin biosynthesis pathway

The reference sets of genes in the anthocyanin biosynthesis pathway were derived from *A. thaliana* [120] except AN2, which was derived from *P. hybrida* [111] and *S. lycopersicum* [112]. The protein sequences were used as queries to align with the protein annotation library of the *L. ruthenicum* genome. The same methods were used to identify homologous genes involved in the anthocyanin biosynthesis pathway in the genome sequence of *L. ruthenicum* [104]. Gene cloning (*LrCHS1*, *LrCHS2*, *LrAOMT*, *LrF3’5’H*, and *LrAN2.1*) and overexpression transient transformation were performed according to the methods of Zhao et al. [104] using young leaves of NQ No.1. Based on the *Lycium* genome and transcriptome data, all the primers for qRT-PCR, gene cloning, and overexpression were designed to amplify the CDS sequence, and are detailed in Table S9.

## Supplementary Material

Web_Material_uhae298

## Data Availability

Data supporting the findings of this work are available within the paper and the Supplementary Tables and Figures. The genome assembly, HiFi reads and HiC data have been deposited into the National Center for Biotechnology Information Sequence Read Archive database with accession numbers JAUDPO000000000, PRJNA1099465 and PRJNA1099464, respectively. The genome assembly data is also available on the Figshare platform (https://doi.org/10.6084/m9.figshare.26550406).
