# Supplementary figures and images for: High-quality genome of black wolfberry (Lycium ruthenicum Murr.) provides insights into the genetics of anthocyanin biosynthesis regulation
Source: Hortic Res. 2024 Oct 23;12(2):uhae298. doi: 10.1093/hr/uhae298 (PMC11822397; doi:10.1093/hr/uhae298)

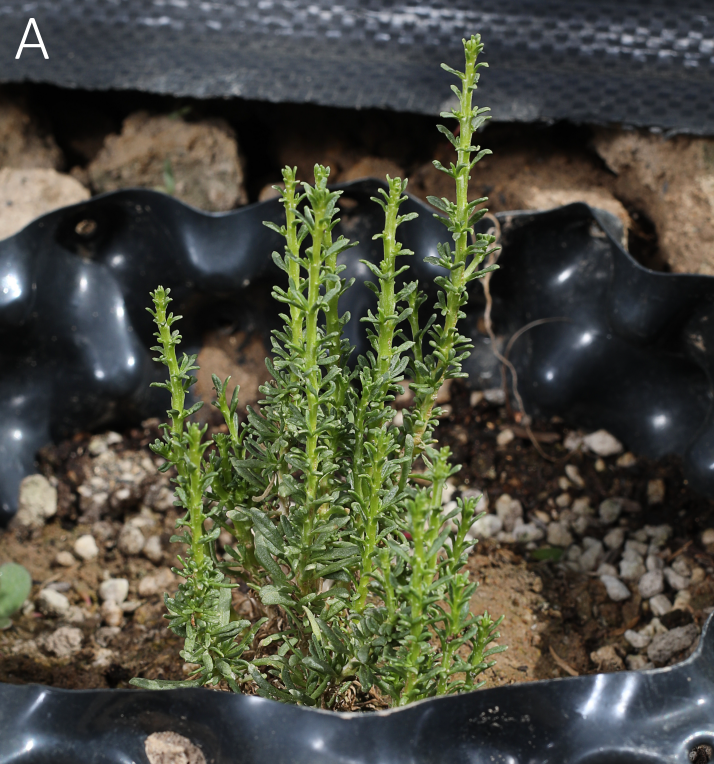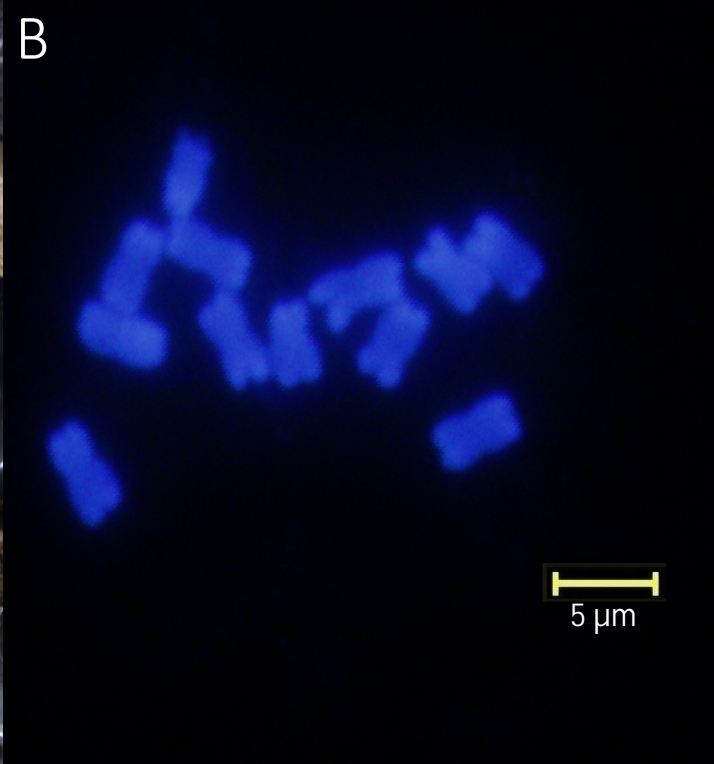

Supplement: Web_Material_uhae298 [file web_material_uhae298.zip › 7.Figure S1. The appearance (A) and chromosome karyotype (B) of black wolfberry accession 'Heiguo'.pdf]

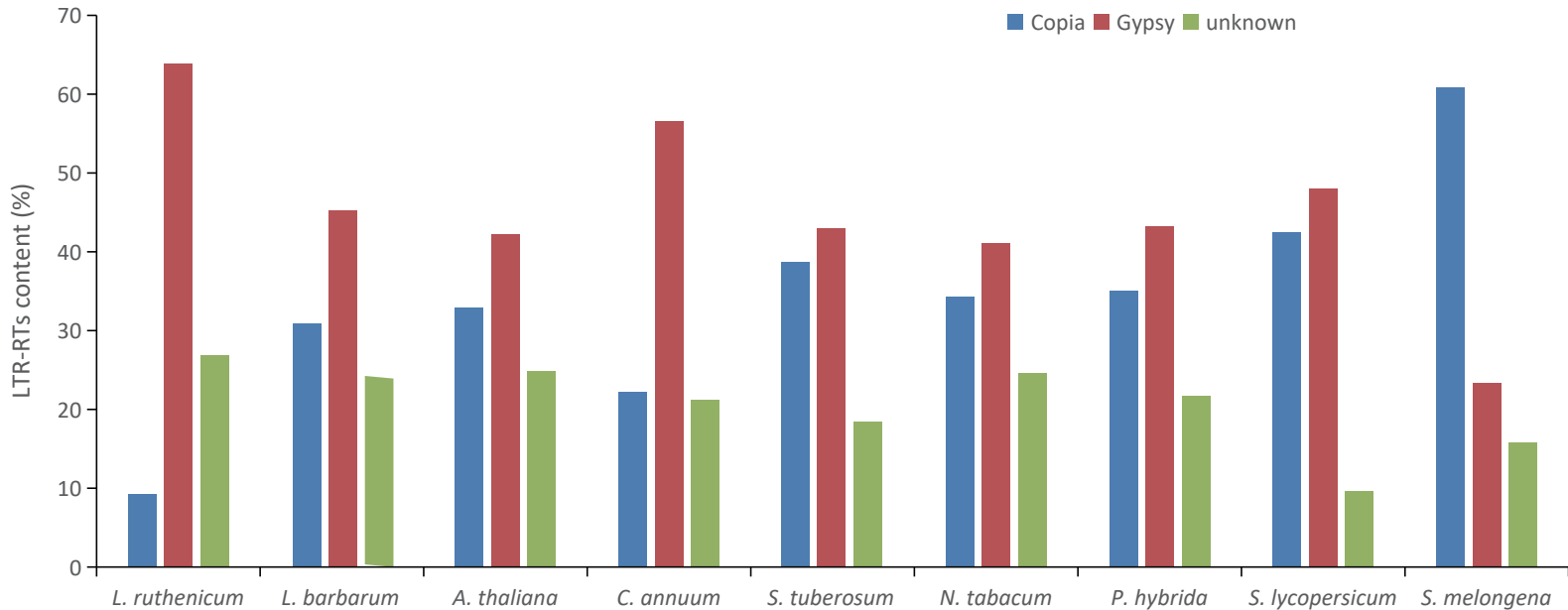

Supplement: Web_Material_uhae298 [file web_material_uhae298.zip › 8.Figure S2.The percentage of three different types of long terminal repeat retrotransposons (LTR-RTs) in the nine species.pdf]

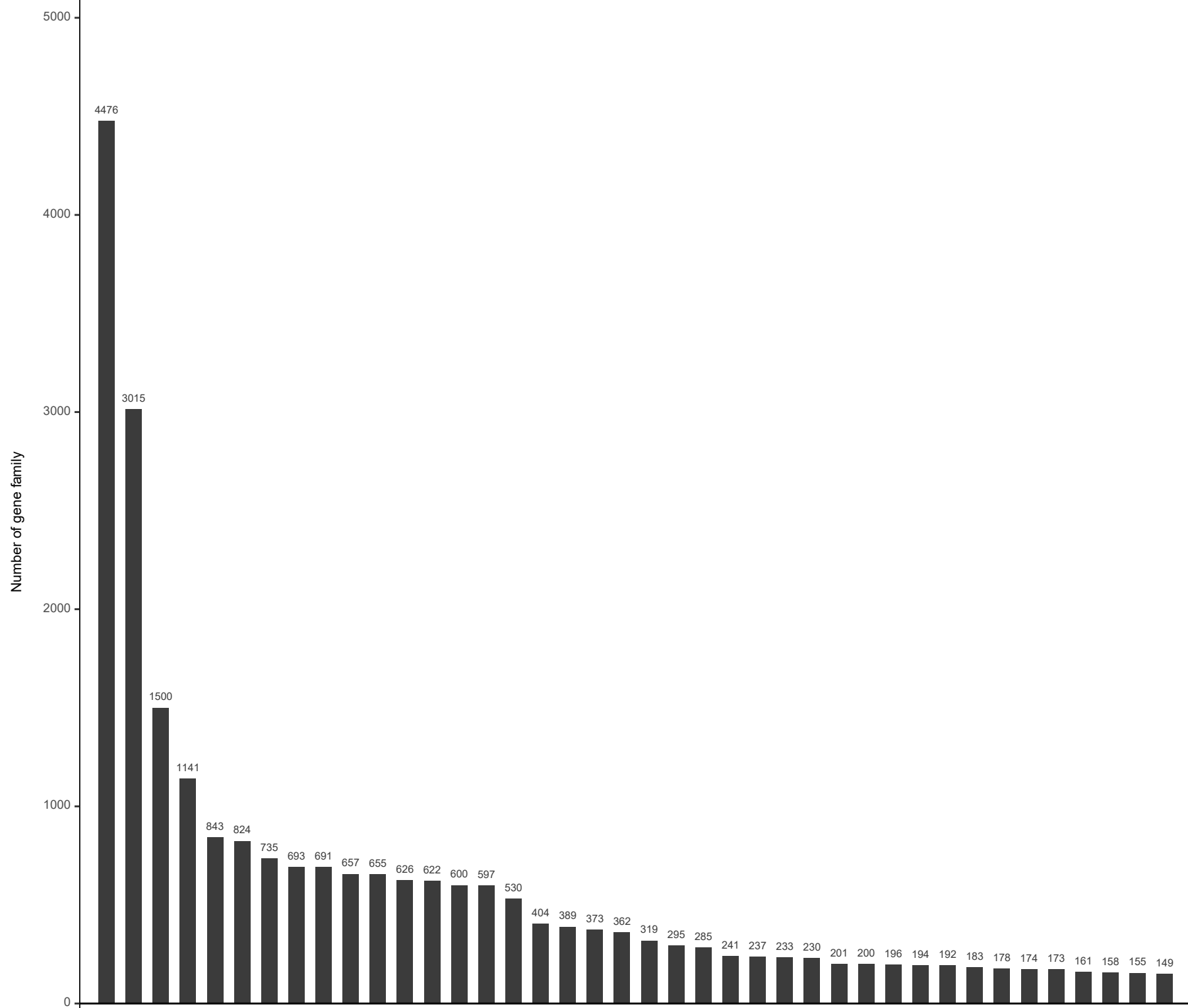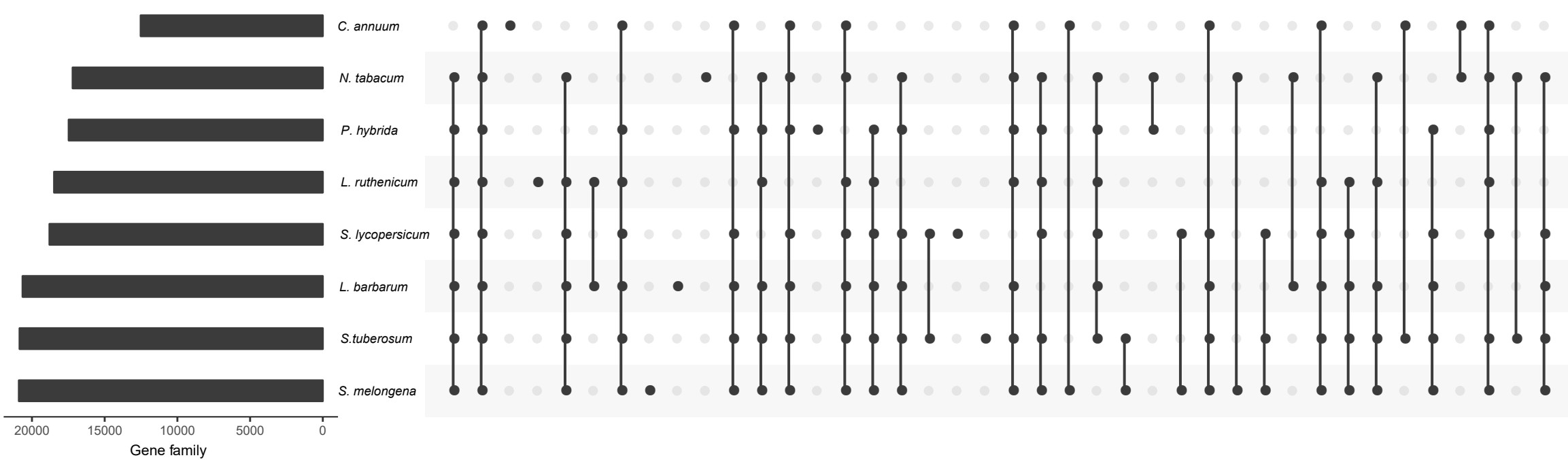

Supplement: Web_Material_uhae298 [file web_material_uhae298.zip › 9.Figure S3.UpSet plot of the gene families.pdf]

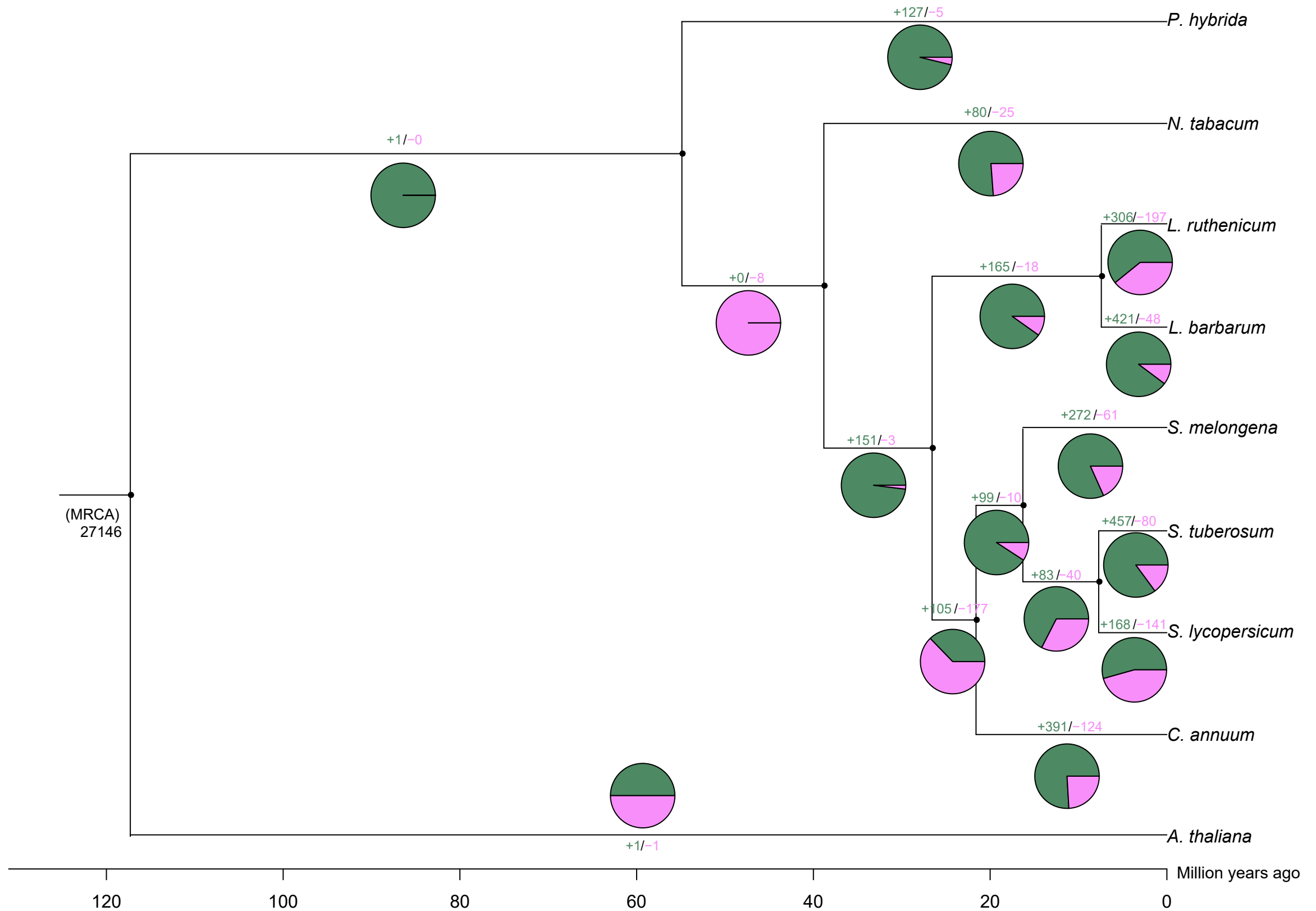

Supplement: Web_Material_uhae298 [file web_material_uhae298.zip › 10.Figure S4. Gene family expansion and contraction analysis.pdf]

# KEGG pathway

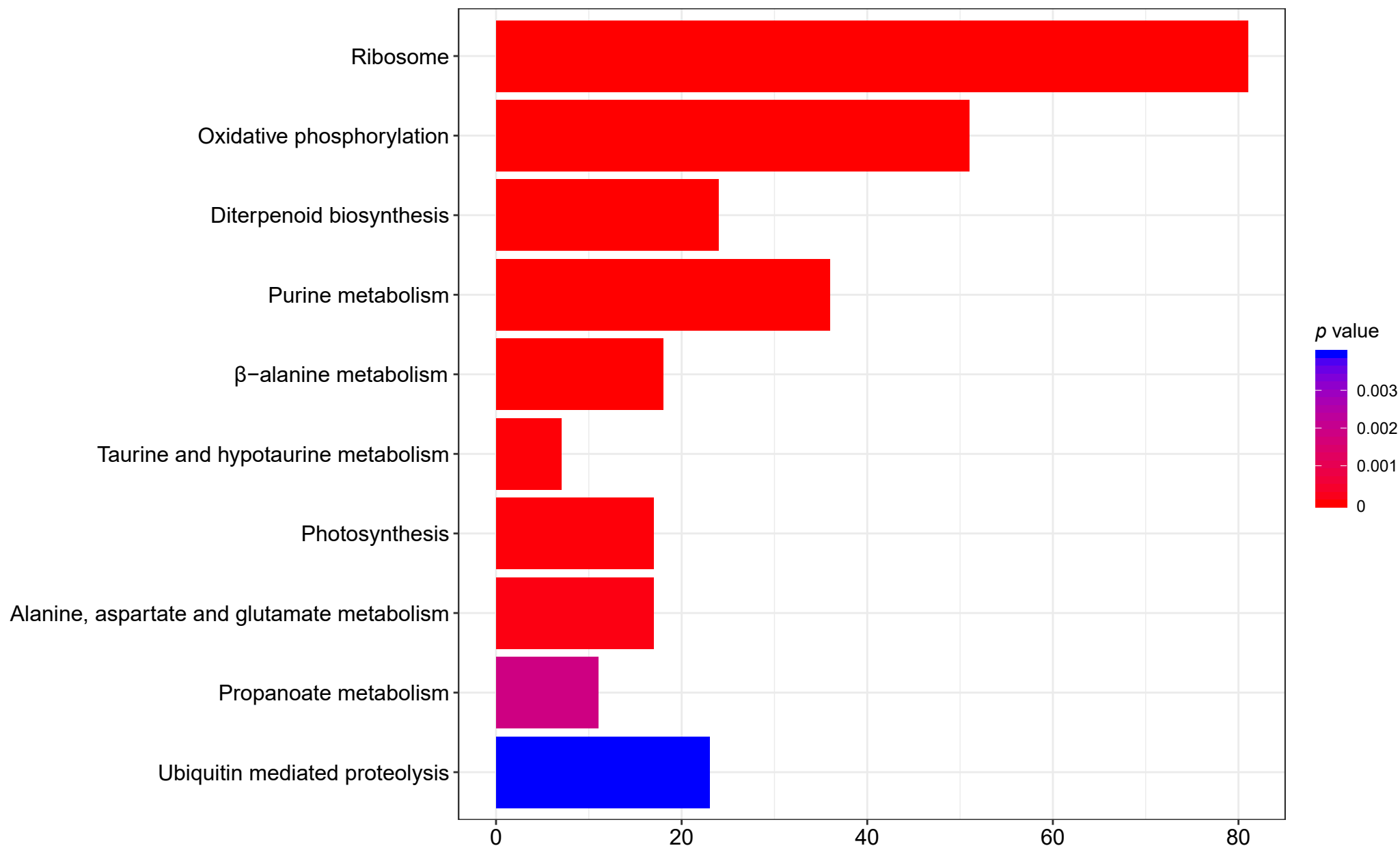

Supplement: Web_Material_uhae298 [file web_material_uhae298.zip › 11.Figure S5. KEGG enrichment analysis based on positively selected genes in the genome of L. ruthenicum.pdf]

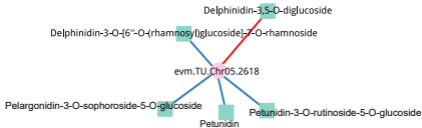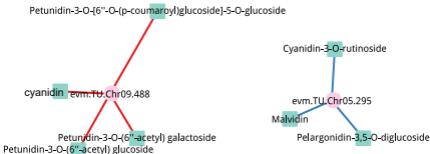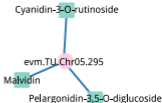

Supplement: Web_Material_uhae298 [file web_material_uhae298.zip › 12.Figure S6. Correlation network diagram of between the differentially expressed anthocyanin biosynthesis pathway genes and anthocyanin-related metabolit.pdf]

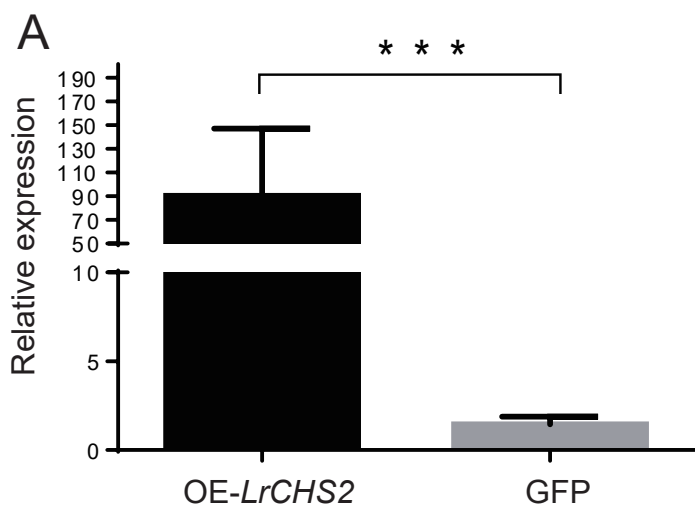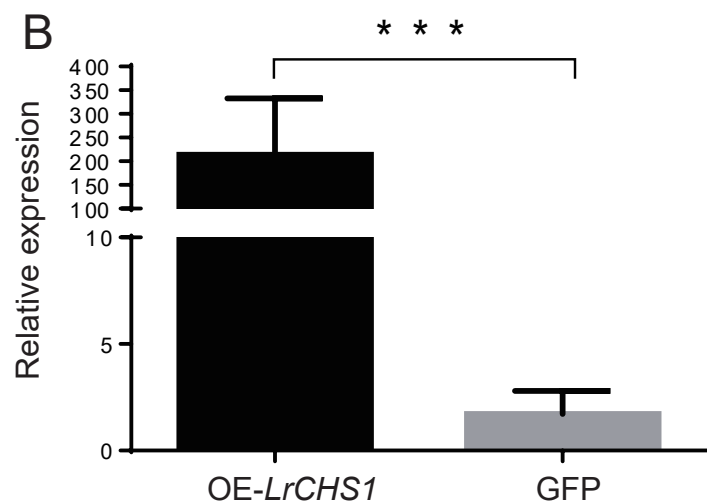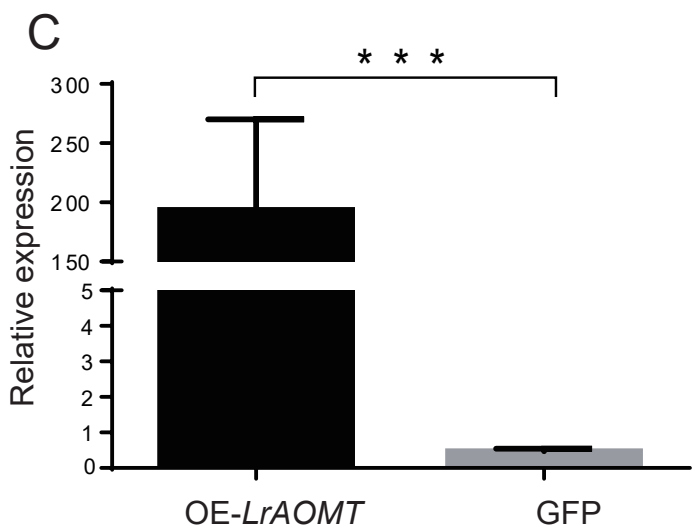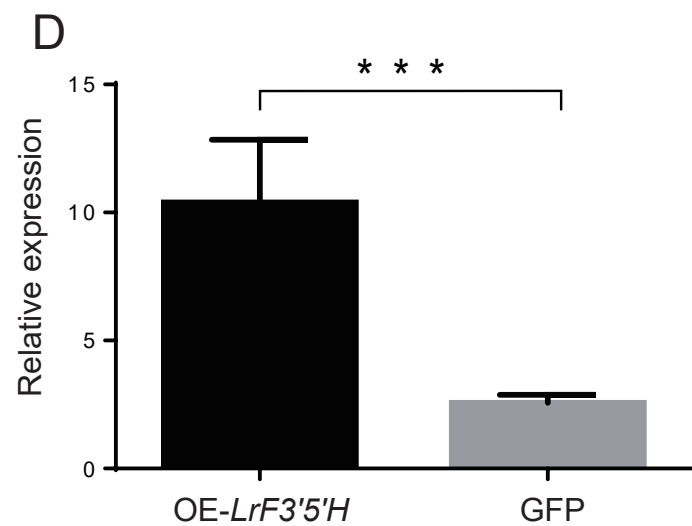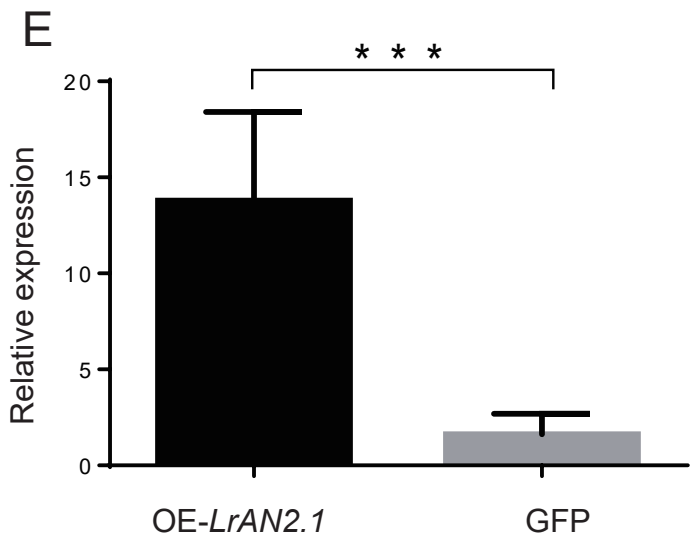

Supplement: Web_Material_uhae298 [file web_material_uhae298.zip › 13.Figure S7. Relative expression of the five genes in OE plants.pdf]

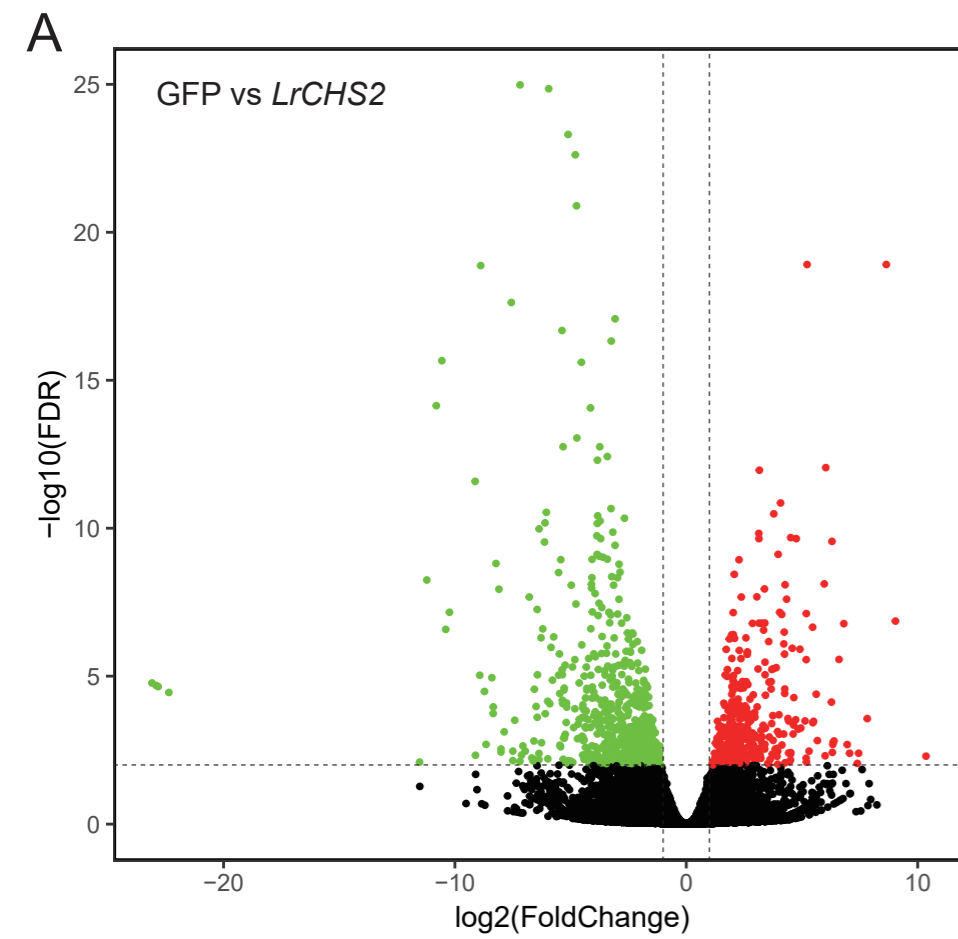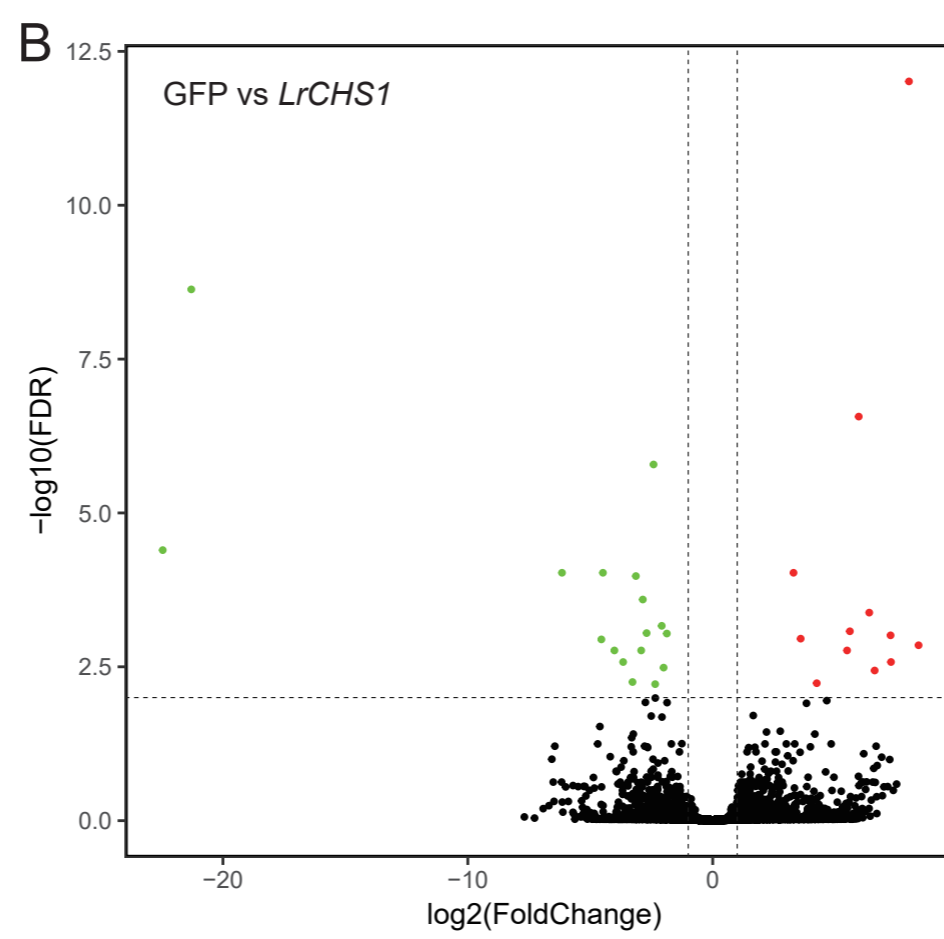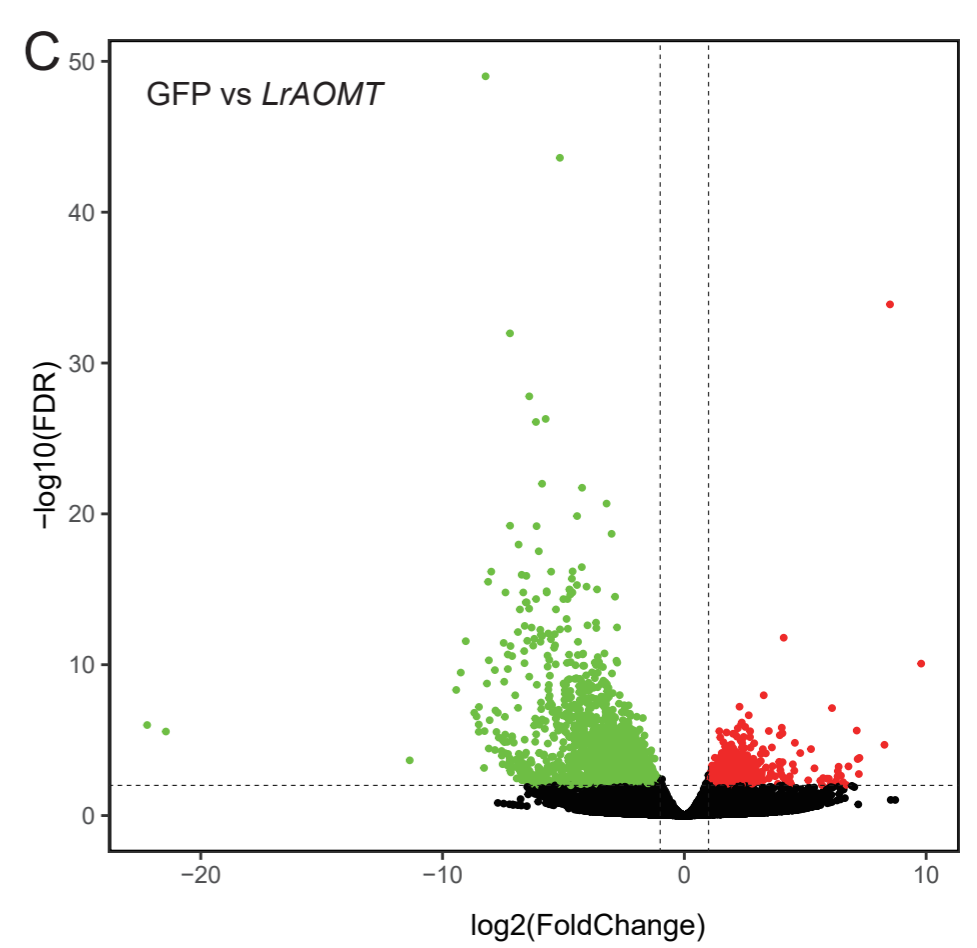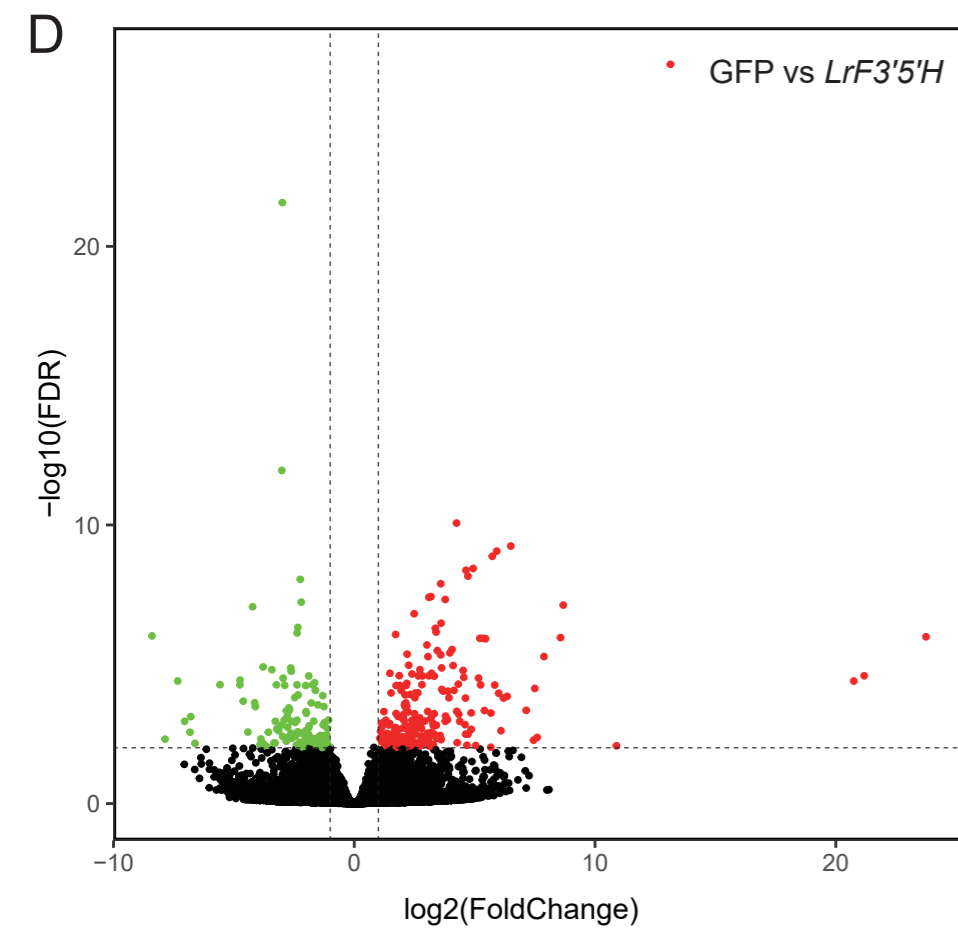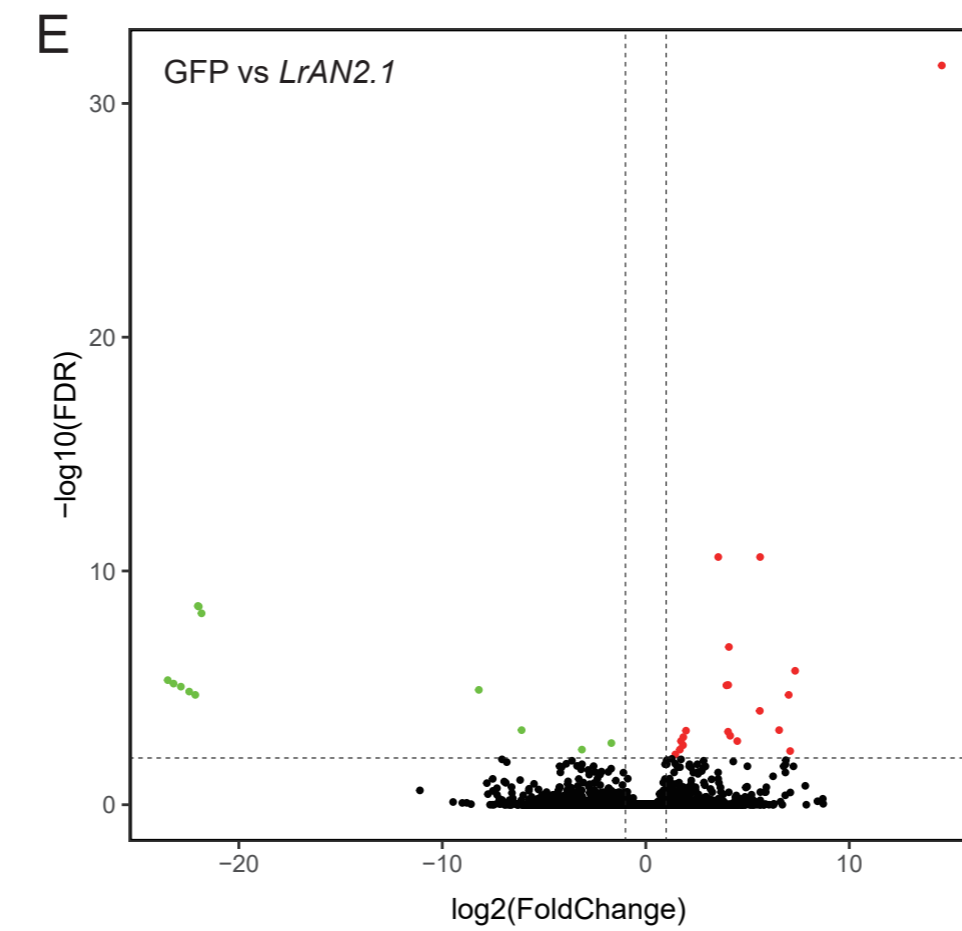

- Up-regulated
- Down-regulated
- No significant change

Supplement: Web_Material_uhae298 [file web_material_uhae298.zip › 14.Figure S8. Volcano plot showing differentially expressed genes (DEGs) between comparisons.pdf]

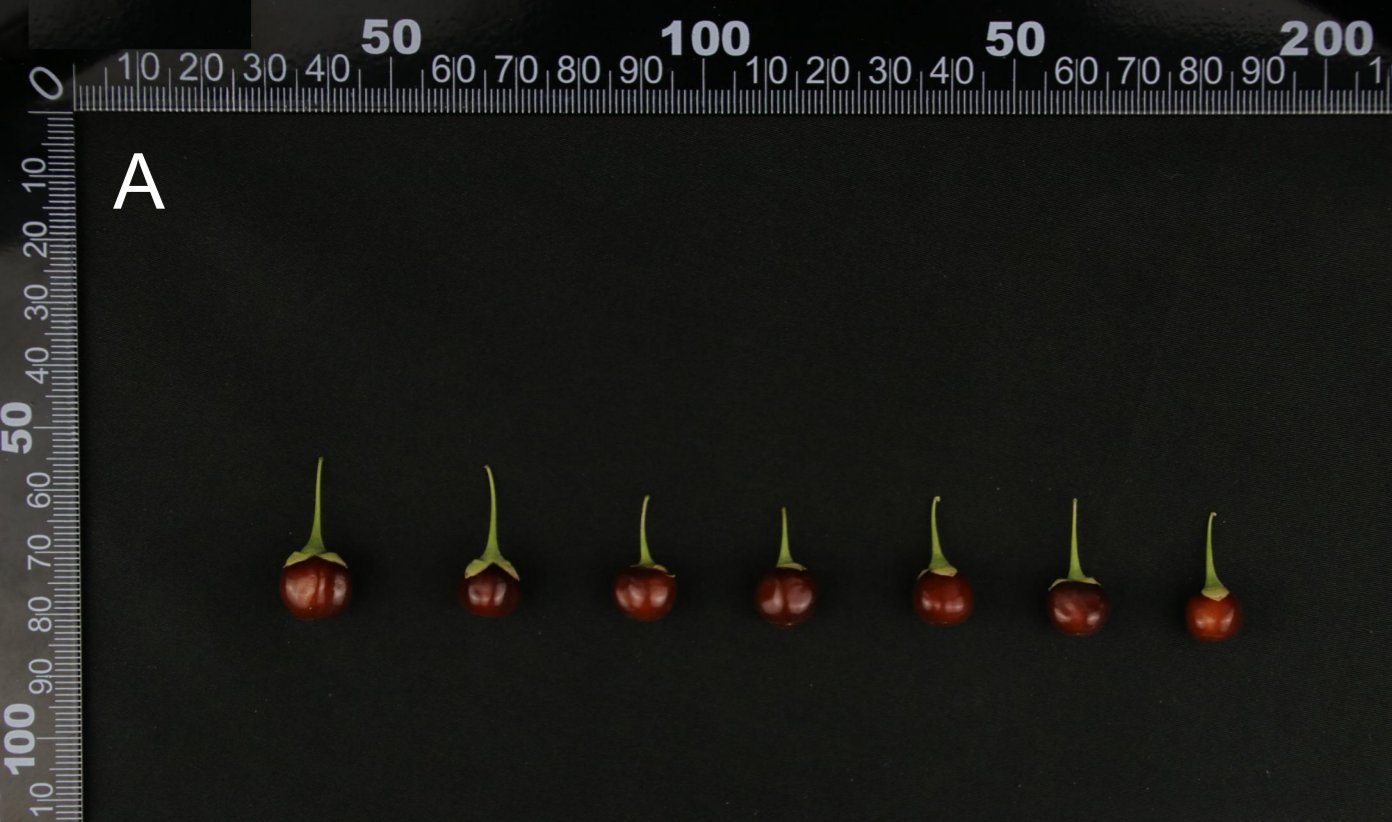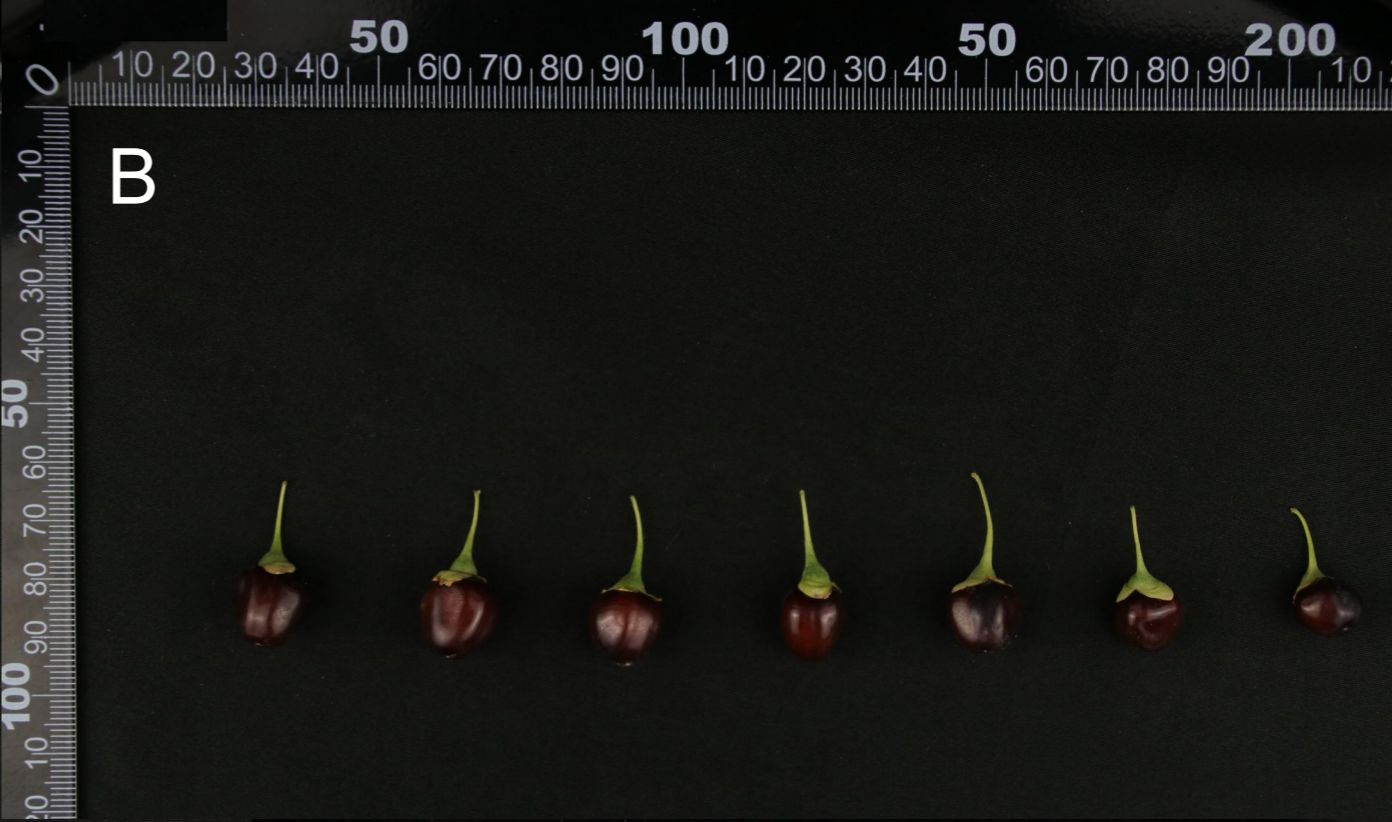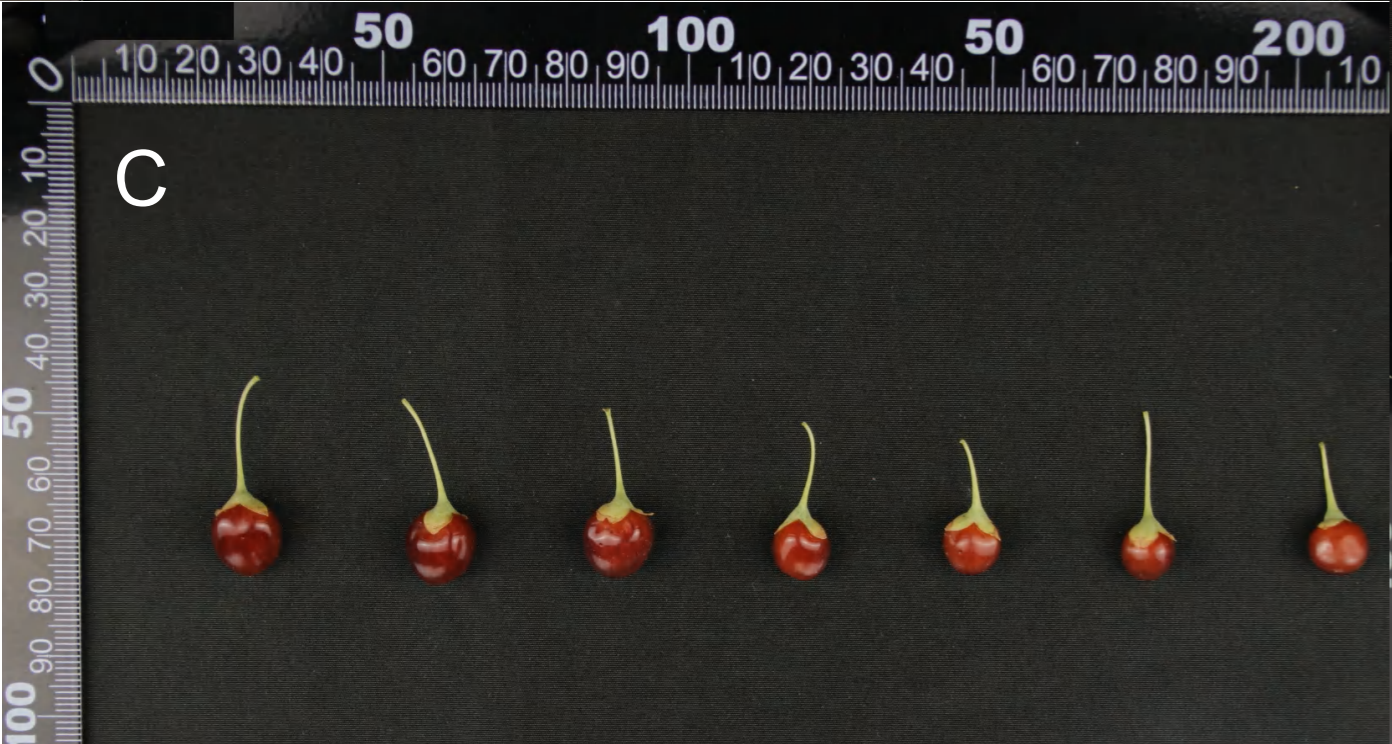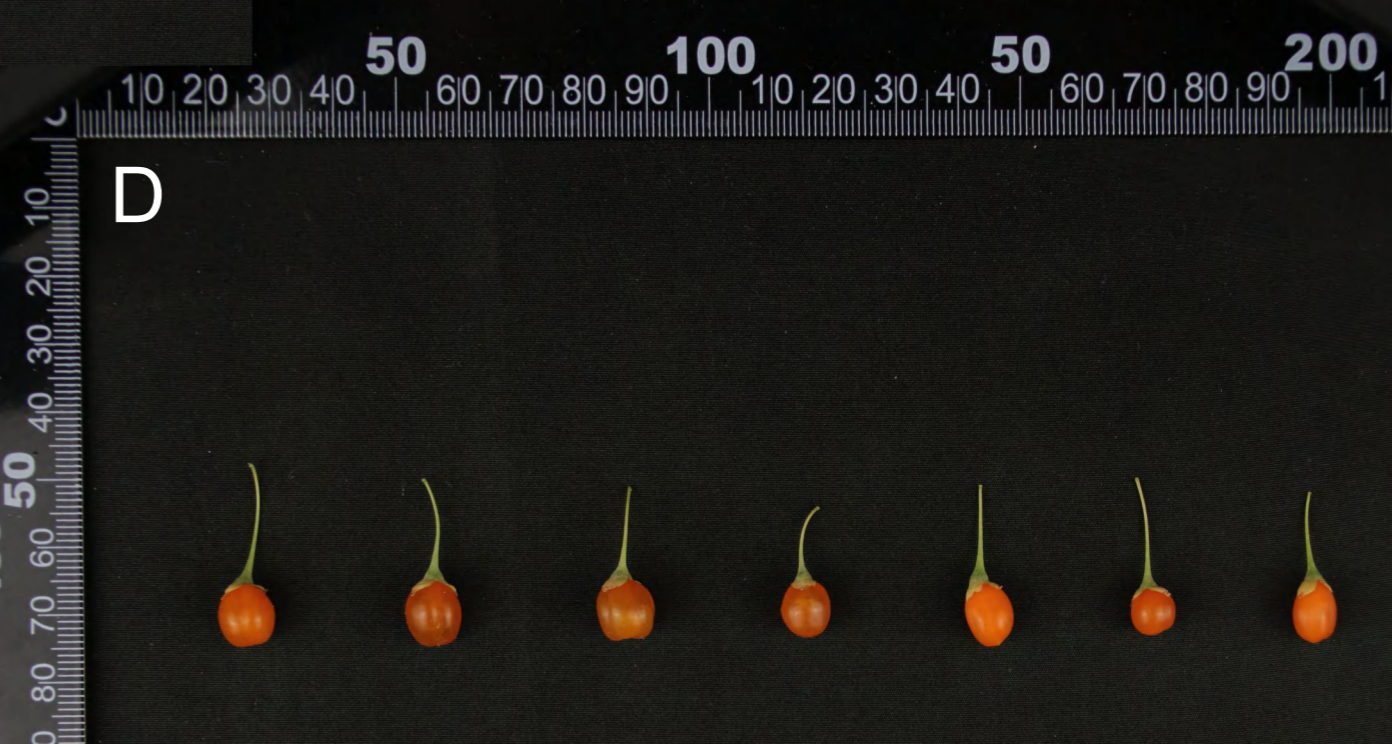

Supplement: Web_Material_uhae298 [file web_material_uhae298.zip › ï1⁄4^å·2åZ<ç1⁄4cï1⁄4%15.Figure S9. The sampling fruits performance of accessions 'HZ-13-01' (A), 'Zhuxi07' (B), 'Zh-13-0802' (C) and 'QH-13-0806' (D).pdf]
